# Supplementary material for: AAV1 is the optimal viral vector for optogenetic experiments in pigeons (Columba livia)
Source: Commun Biol. 2021 Jan 22;4:100. doi: 10.1038/s42003-020-01595-9 (PMC7822860; doi:10.1038/s42003-020-01595-9)
Supplement: Supplementary file 2 — Supplementary Information [file 42003_2020_1595_MOESM2_ESM.pdf]

AAV1 is the optimal viral vector for optogenetic experiments in pigeons *(Columba livia)*

Noemi Rook[1#], John Michael Tuff[1], Sevim Isparta[1,2], Olivia Masseck[3] Stefan Herlitze[4], Onur Güntürkün[1], Roland Pusch[1]

[1] Department of Biopsychology, Institute of Cognitive Neuroscience, Faculty of Psychology, Ruhr University Bochum, Universitätsstraße 150, 44801 Bochum, Germany
[2]Department of Genetics, Faculty of Veterinary Medicine, Ankara University, Şht. Ömer Halisdemir Blv, 06110 Ankara, Turkey
[3]University of Bremen, Synthetic Biology, Leobener Straße 5, 28359 Bremen, Germany
[4]Department of General Zoology and Neurobiology, Ruhr University Bochum, Universitätsstraße 150, 44801 Bochum, Germany

Address for correspondence
Noemi Rook
Biopsychology, Faculty of Psychology, Ruhr University Bochum, Germany
Universitätsstraße 150, 44780 Bochum, Germany
Phone: +49 234 32 21628
E-mail: noemi.rook@rub.de

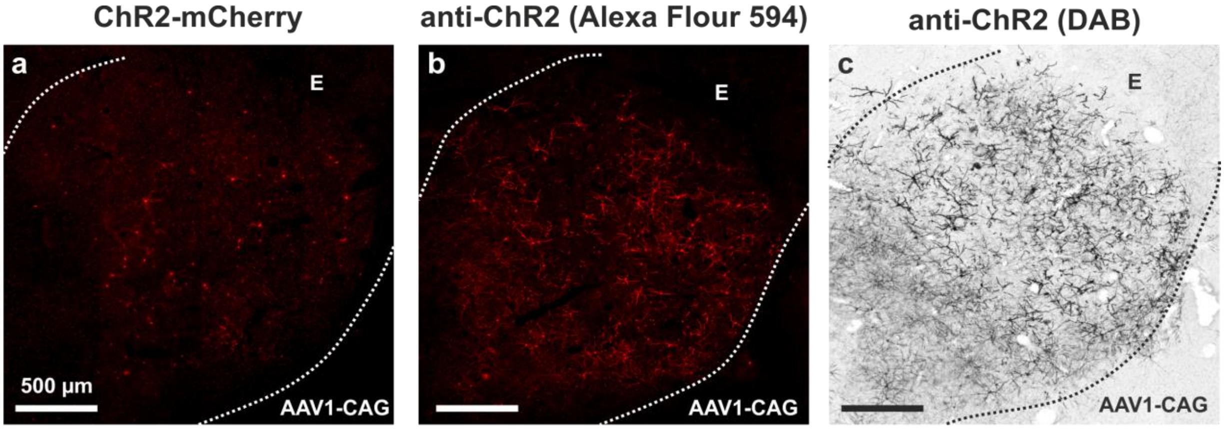

Supplementary Fig. 1. Detectable transgene expression increases with immunohistochemical stainings. (a-c) Microscopic images within the entopallium of the same pigeon in different brain series depicting: **(a)** native ChR2-mCherry expression following injections of AAV1-CAG-ChR2-mCherry. **(b)** ChR2 expression following injections of AAV1-CAG-ChR2-mCherry combined with an immunohistochemical staining against ChR2 with Alexa Flour 594(red). **(c)** ChR2 expression following injections of AAV1-CAG-ChR2-mCherry combined with an immunohistochemical staining against ChR2 and a subsequent 3,3' diaminobenzidine (DAB) reaction. The amount of detectable ChR2 expressing cells is greater for the counterstained sections. All scale bars represent 500 µm.

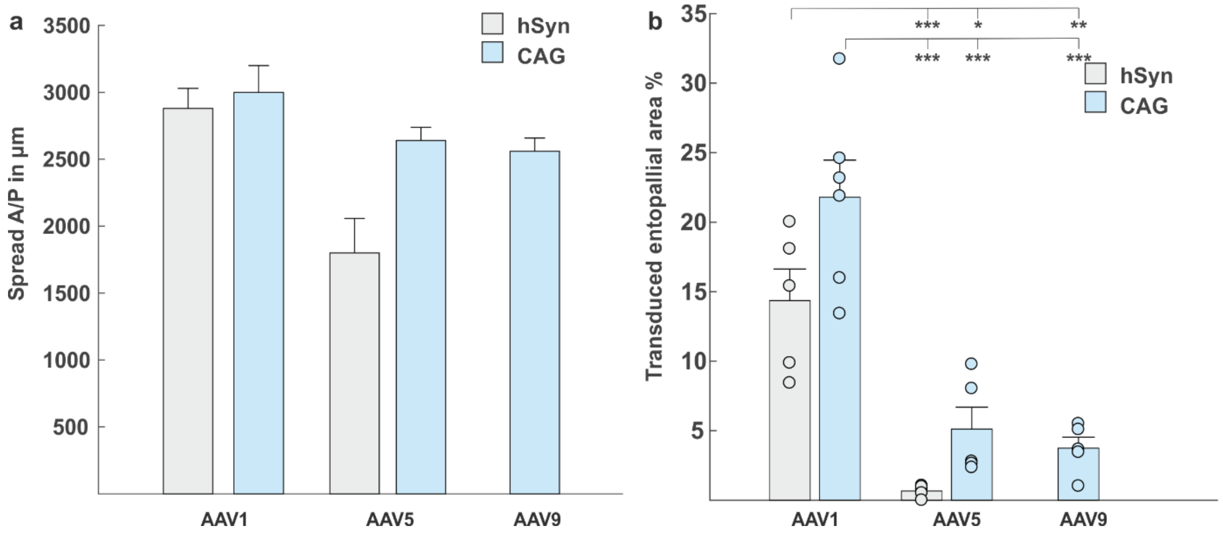

Supplementary Fig. 2. Transduction efficiencies of all tested AAVs. (a) Spread of all serotypes in the anterior posterior plane within the entopallium. **(b)** Percentage of ChR2 expressing area compared to the size of the entopallium in slices with transduction. The AAVs with the hSyn promoter a depicted in grey and with the CAG promoter in blue. Error bars depict the standard error of the mean (SEM), dots represent raw data, ***p < .001, **p <. 01, *p < .05.

Supplementary Table 1. Classification of anterograde and retrograde transgene expression in NI/MVL, striatum and nucleus rotundus for all cases of AAV1-CAG and AAV1-hSyn injections into the entopallium. Cases were classified into + little, ++ moderate and +++ extensive based on the intensity of transgene expression (see method section for more details).

Case	Serotype	Time	Anterograde NI/MVL	Anterograde Striatum	Retrograde nRT	Supp. Figure
Case 1	AAV1-CAG	> 2 months	+++	+++	+++	S. Fig. 3,5,7
Case 2	AAV1-CAG	> 2 months	+++	+++	+++	S. Fig. 3,5,7
Case 3	AAV1-CAG	> 2 months	+++	+++	+++	S. Fig. 3,5,7
Case 4	AAV1-CAG	> 2 months	+++	+++	+++	S. Fig. 3,5,7
Case 5	AAV1-CAG	> 2 months	++	+	+++	S. Fig. 3,5,7
Case 6	AAV1-CAG	> 2 months	++	+++	++	S. Fig. 3,5,7
Case 7	AAV1-CAG	> 2 months	+++	+++	+++	S. Fig. 3,5,7
Case 8	AAV1-CAG	> 2 months	+++	+++	+++	S. Fig. 3,5,7
Case 9	AAV1-CAG	6 weeks	++	+	+++	S. Fig. 3,5,7
Case 10	AAV1-CAG	6 weeks	+	+	++	S. Fig. 3,5,7
Case 11	AAV1-CAG	6 weeks	++	+	+	S. Fig. 3,5,7
Case 12	AAV1-CAG	6 weeks	+	++	+	S. Fig. 3,5,7
Case 13	AAV1-CAG	6 weeks	++	++	n.a.	S. Fig. 3,5
Case 14	AAV1-CAG	6 weeks	++	+	n.a.	S. Fig. 3,5
Case 15	AAV1-hSyn	> 2 months	+	+	+	S. Fig. 4,6,8
Case 16	AAV1-hSyn	> 2 months	+	+	+	S. Fig. 4,6,8
Case 17	AAV1-hSyn	> 2 months	+	+	+	S. Fig. 4,6,8
Case 18	AAV1-hSyn	> 2 months	+	+	+	S. Fig. 4,6,8
Case 19	AAV1-hSyn	6 weeks	+	+	+	S. Fig. 4,6,8
Case 20	AAV1-hSyn	6 weeks	+	+	+	S. Fig. 4,6,8
Case 21	AAV1-hSyn	6 weeks	+	+	++	S. Fig. 4,6,8
Case 22	AAV1-hSyn	6 weeks	+	++	++	S. Fig. 4,6,8
Case 23	AAV1-hSyn	6 weeks	+	+	+	S. Fig. 4,6,8
Case 24	AAV1-hSyn	6 weeks	+	+	+	S. Fig. 4,6,8

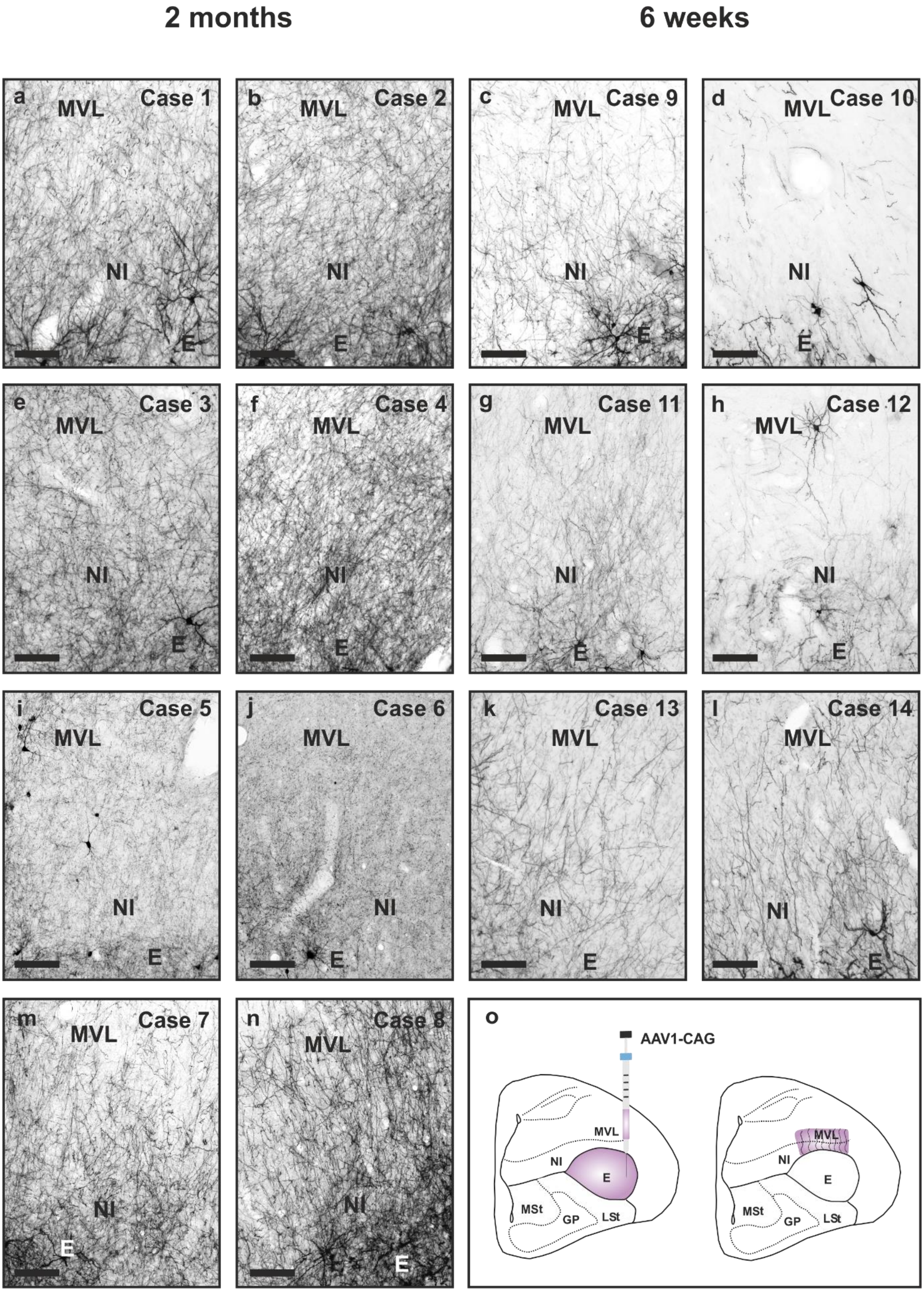

Supplementary Fig. 3. Anterograde ChR2 expression in NI/MVL following injections of AAV1-CAG-ChR2 into the entopallium. Sections were stained against ChR2 and the signal was visualized with a DAB staining procedure. Cases 1 - 8 had longer expression times (> 2 months) and cases 9-14 were perfused 6 weeks after the injections. All scale bars depict 100 µm.

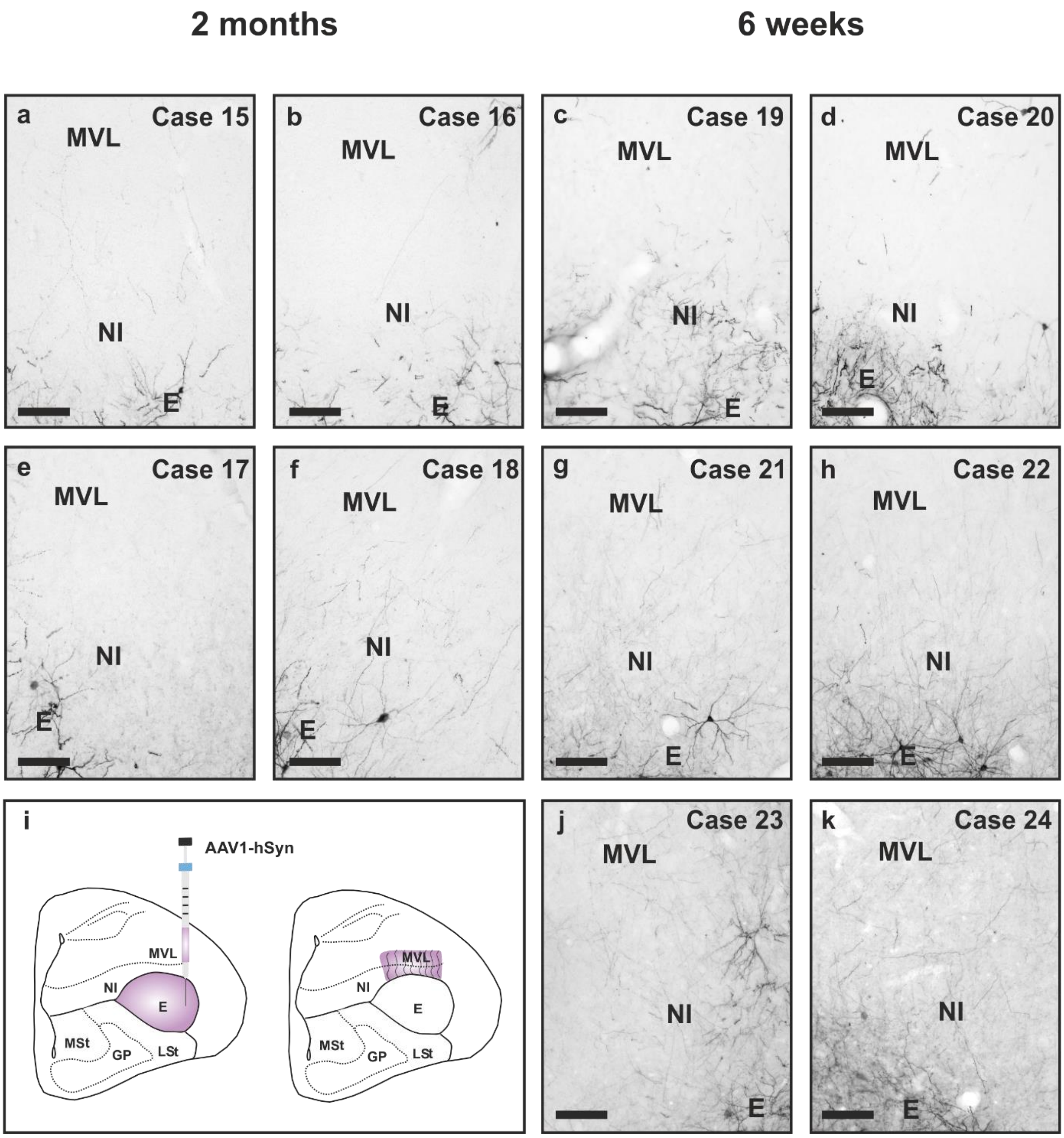

Supplementary Fig. 4. Anterograde ChR2 expression in NI/MVL following injections of AAV1-hSyn-ChR2 into the entopallium. Sections were stained against ChR2 and the signal was visualized with a DAB staining procedure. Cases 15 - 18 had longer expression times (> 2 months) and cases 19-24 were perfused 6 weeks after the injections. All scale bars depict 100 µm.

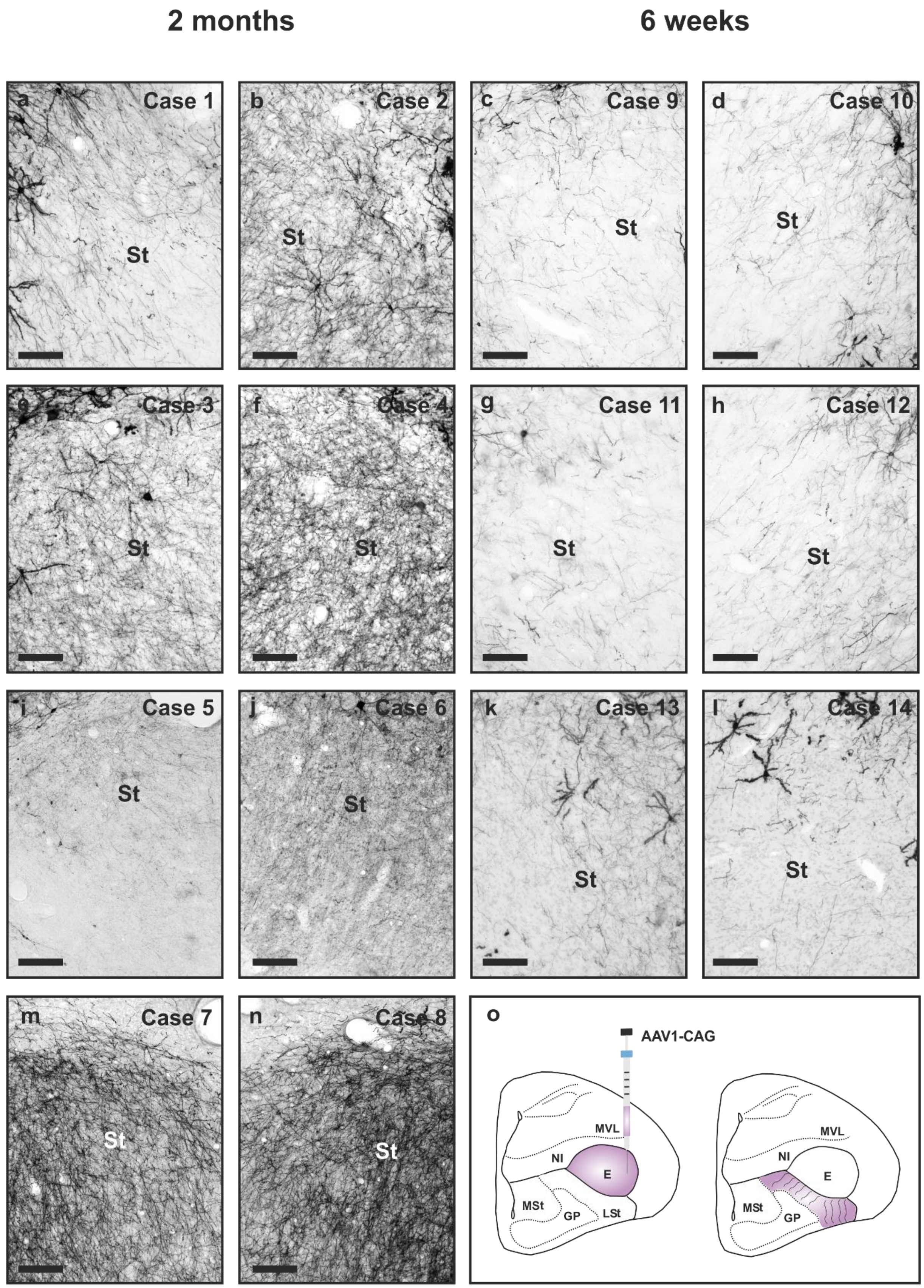

Supplementary Fig. 5. Anterograde ChR2 expression in striatum following injections of AAV1-CAG-ChR2 into the entopallium. Sections were stained against ChR2 and the signal was visualized with a DAB staining procedure. Cases 1 - 8 had longer expression times (> 2 months) and cases 9-14 were perfused 6 weeks after the injections. All scale bars depict 100 µm.

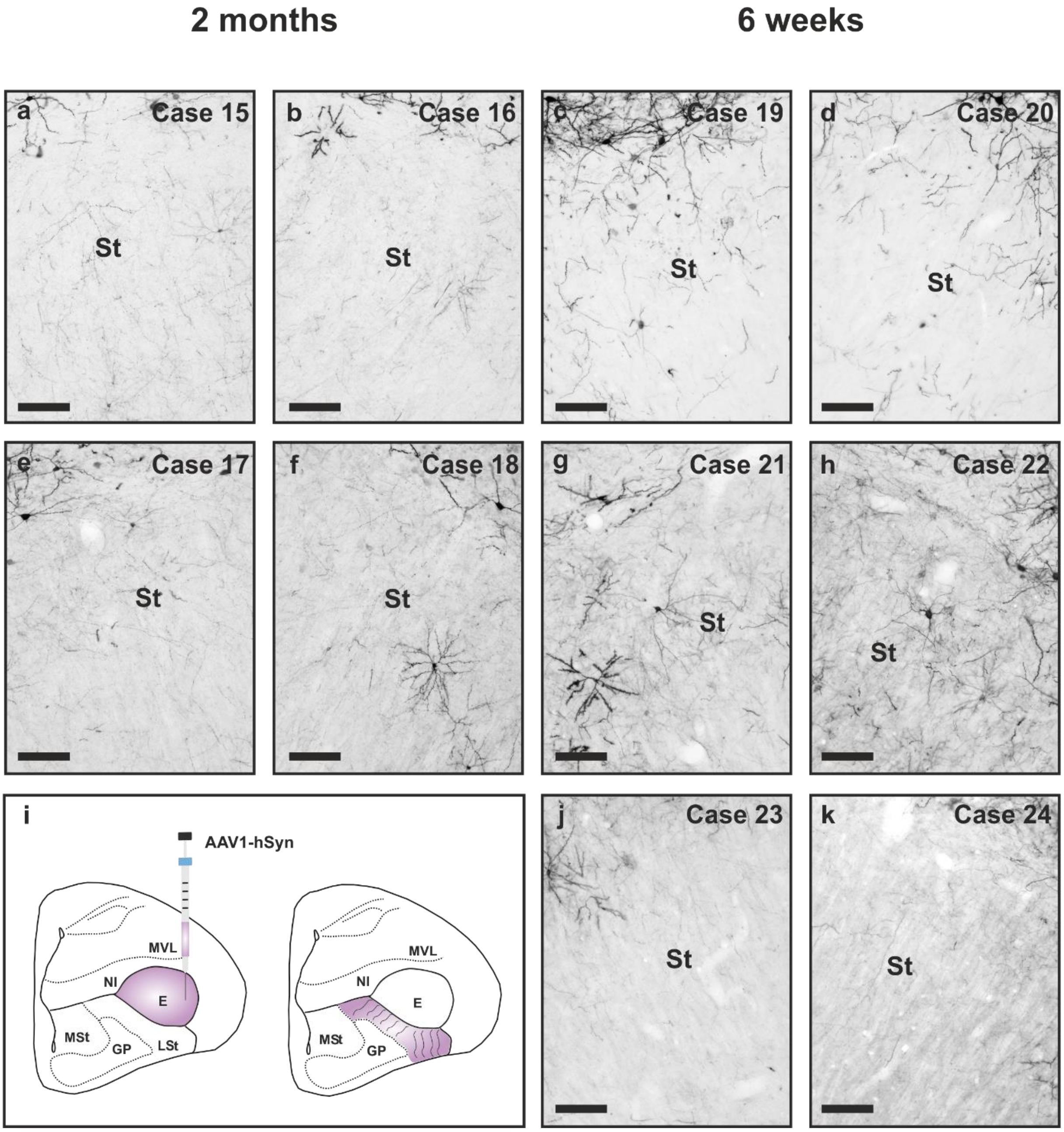

Supplementary Fig. 6. Anterograde ChR2 expression in striatum following injections of AAV1-hSyn-ChR2 into the entopallium. Sections were stained against ChR2 and the signal was visualized with a DAB staining procedure. Cases 15 - 18 had longer expression times (> 2 months) and cases 19-24 were perfused 6 weeks after the injections. All scale bars depict 100 µm.

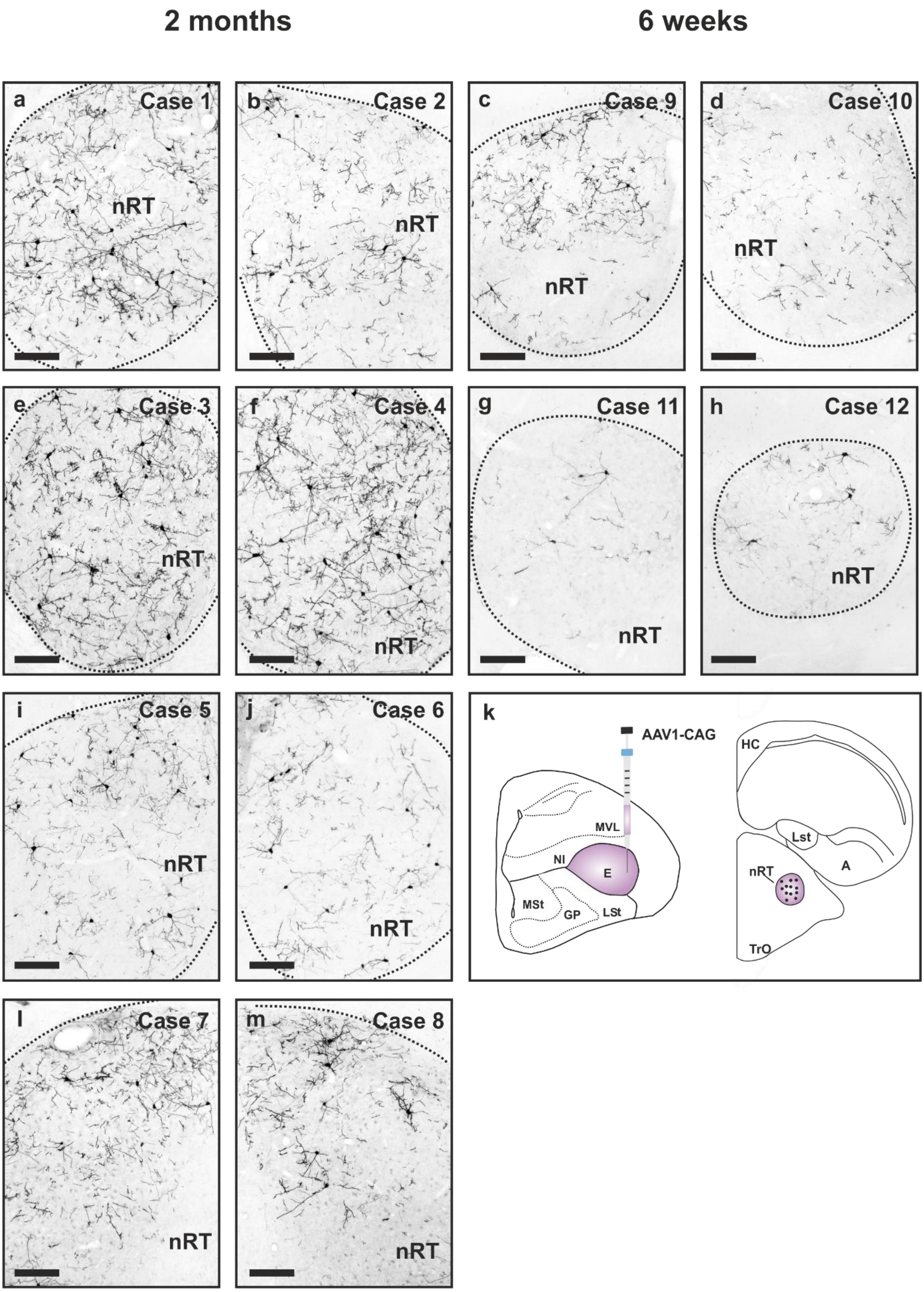

Supplementary Fig. 7. Retrograde ChR2 expression in the nucleus rotundus following injections of AAV1-CAG-ChR2 into the entopallium. Sections were stained against ChR2 and the signal was visualized with a DAB staining procedure. Cases 1 - 8 had longer expression times (> 2 months) and cases 9-12 were perfused 6 weeks after the injections. All scale bars depict 100 µm

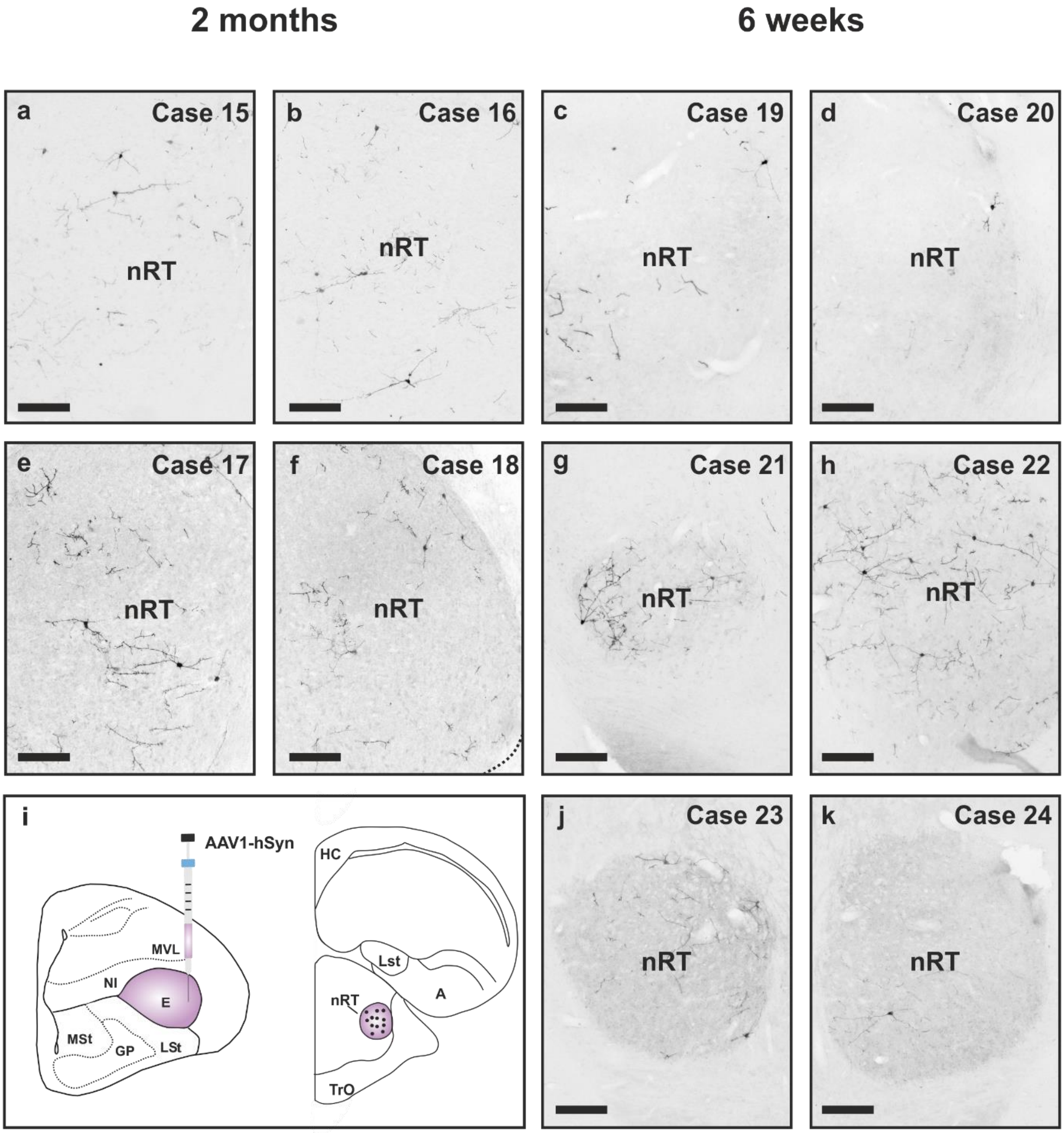

Supplementary Fig. 8. Retrograde ChR2 expression in the nucleus rotundus following injections of AAV1-hSyn-ChR2 into the entopallium. Sections were stained against ChR2 and the signal was visualized with a DAB staining procedure. Cases 15 - 18 had longer expression times (> 2 months) and cases 19-24 were perfused 6 weeks after the injections. All scale bars depict 100 µm.

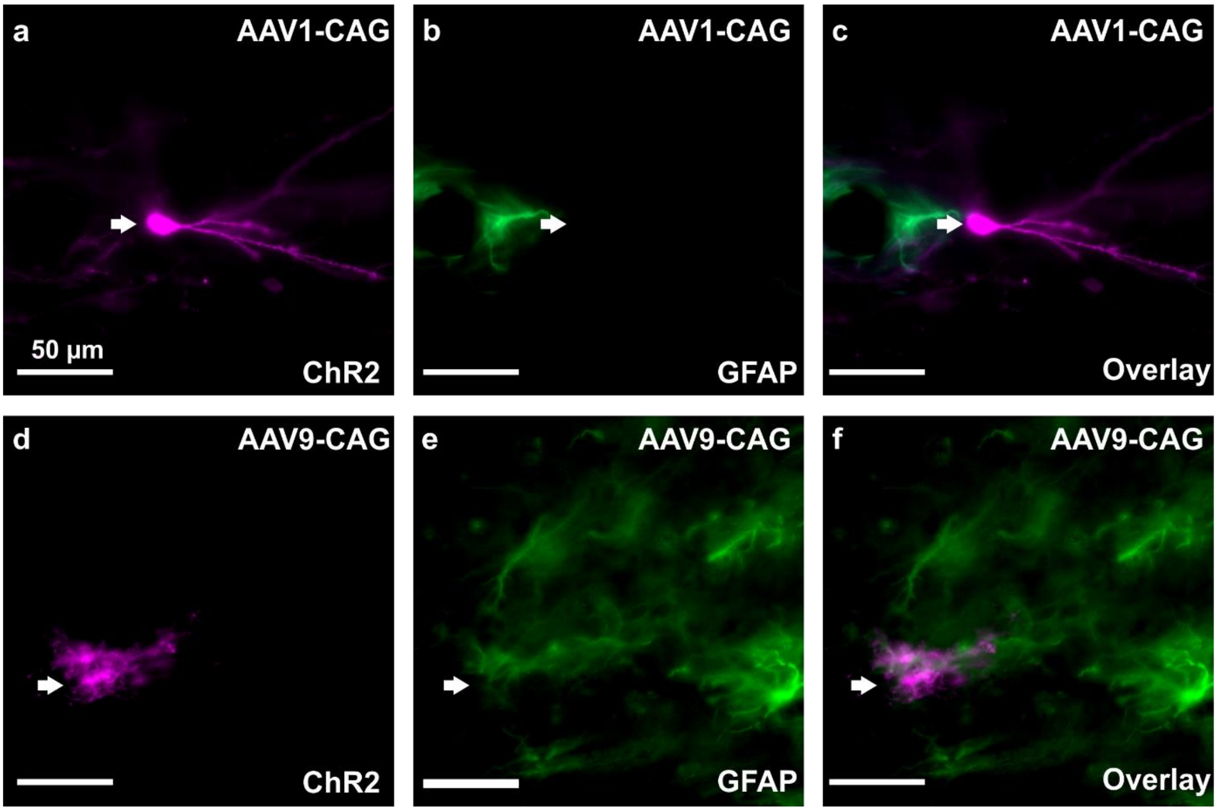

Supplementary Fig. 9. Assessment of glial activation/ inflammatory response. (a) ChR2 expression following injections of AAV1-CAG-ChR2. **(b)** GFAP expression occurs mainly around blood vessels **(c)** Overlay of ChR2 and GFAP expression **(d)** ChR2 expression following injections of AAV9-CAG-ChR2. **(e)** extensive GFAP expression within the injection site following injections of AAV9-CAG-ChR2. **(f)** Overlay of ChR2 and GFAP expression. All scale bars represent 50 µm.

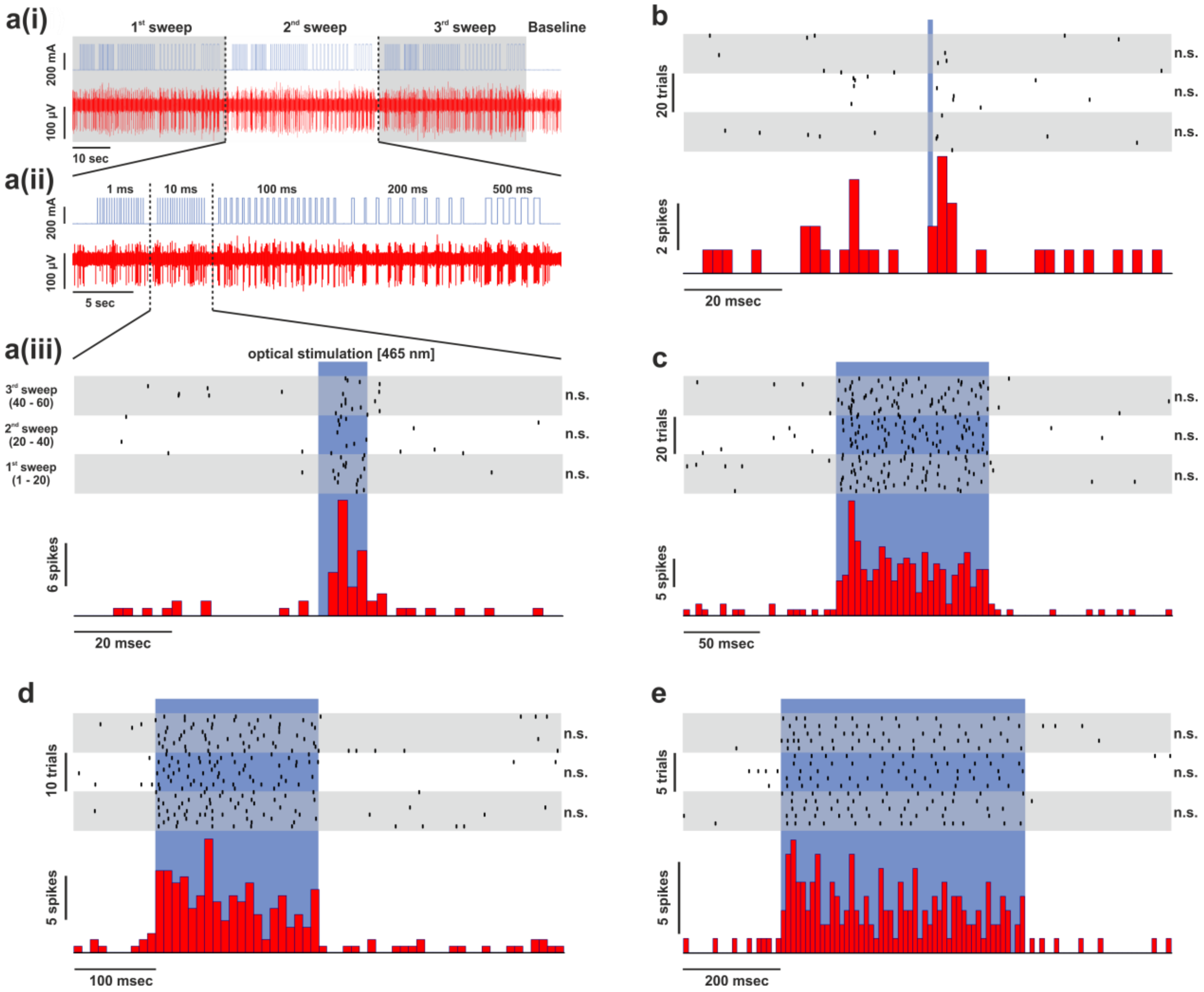

Supplementary Fig. 10. Single cell responses upon optical stimulation (Cell 3). (a(i)) Stimulation protocol (upper trace) and the resulting evoked cellular responses (lower trace). The stimulation protocol consisted of repeated light pulses of different durations (1 ms, 10 ms, 100 ms, 200 ms and 500 ms) and was repeated three times (sweep 1-3, indicated by the gray/white background). The baseline-firing rate of each neuron was assessed before/after the light stimulation protocol. **(a(ii))** A single sweep (i.e. sweep 2) of the optical stimulation protocol. The 10 ms optical stimulation is highlighted and further analyzed in **(a(iii))**. Raster plot (upper part) and peri-stimulus time histogram (PSTH; lower part) of the cell response. The raster plot characterizes the cellular response aligned with the onset of the optical stimulation (465 nm at 300 mA; blue shaded area). Each line represents one optical stimulation. Each dot within that line represents an evoked action potential. For each repetition of the stimulation, a new line is added to the plot. The gray/white background of the raster plot indicates the blocks of repetition (sweep 1-3). To assess the variability of the evoked responses, the sweeps were statistically compared. In the case of this cell, no statistical differences were found (indicated by the abbreviation n.s. next to the raster plots). The PSTH represents the summed responses within a certain time window (bin). In case of the 10 ms stimulus presentation the bin width is 2 ms. **(b)** Raster plot (upper trace) and PSTH (lower trace) for the 1 ms stimulus duration (bin width: 2 ms). **(c)** Raster plot (upper trace) and PSTH (lower trace) for the 100 ms stimulus duration (bin width: 4 ms). **(d)** Raster plot (upper trace) and PSTH (lower trace) for the 200 ms stimulus duration (bin width: 10 ms). **(e)** Raster plot (upper trace) and PSTH (lower trace) for the 500 ms stimulus duration (bin width: 10 ms).

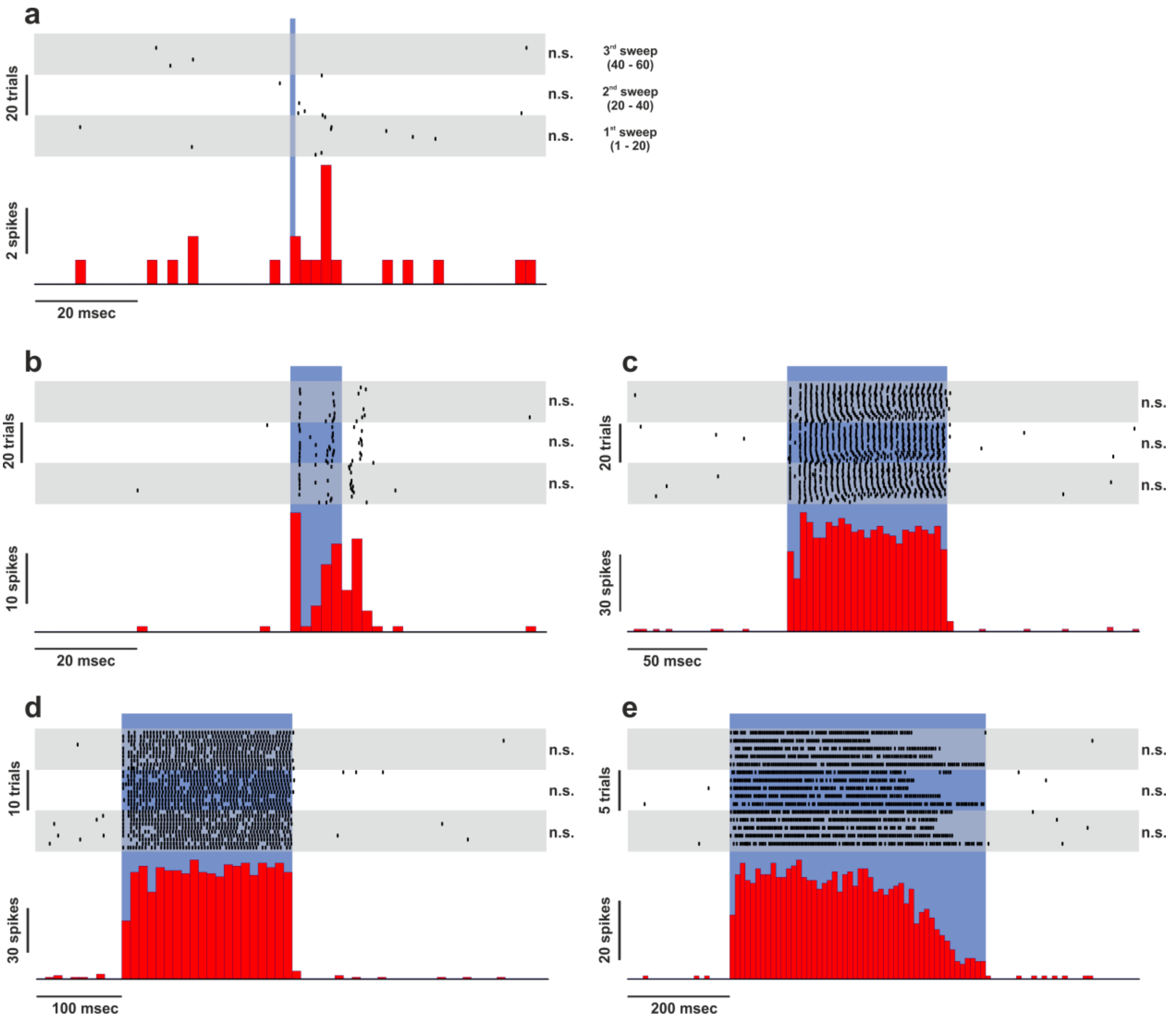

Supplementary Fig. 11. Single cell responses upon optical stimulation (Cell 1). (a) Raster plot (upper trace) and PSTH (lower trace) for the 1 ms stimulus duration (bin width: 2 ms). **(b)** Raster plot (upper trace) and PSTH (lower trace) for the 10 ms stimulus duration (bin width: 2 ms). **(c)** Raster plot (upper trace) and PSTH (lower trace) for the 100 ms stimulus duration (bin width: 4 ms). **(d)** Raster plot (upper trace) and PSTH (lower trace) for the 200 ms stimulus duration (bin width: 10 ms). **(e)** Raster plot (upper trace) and PSTH (lower trace) for the 500 ms stimulus duration (bin width: 10 ms).

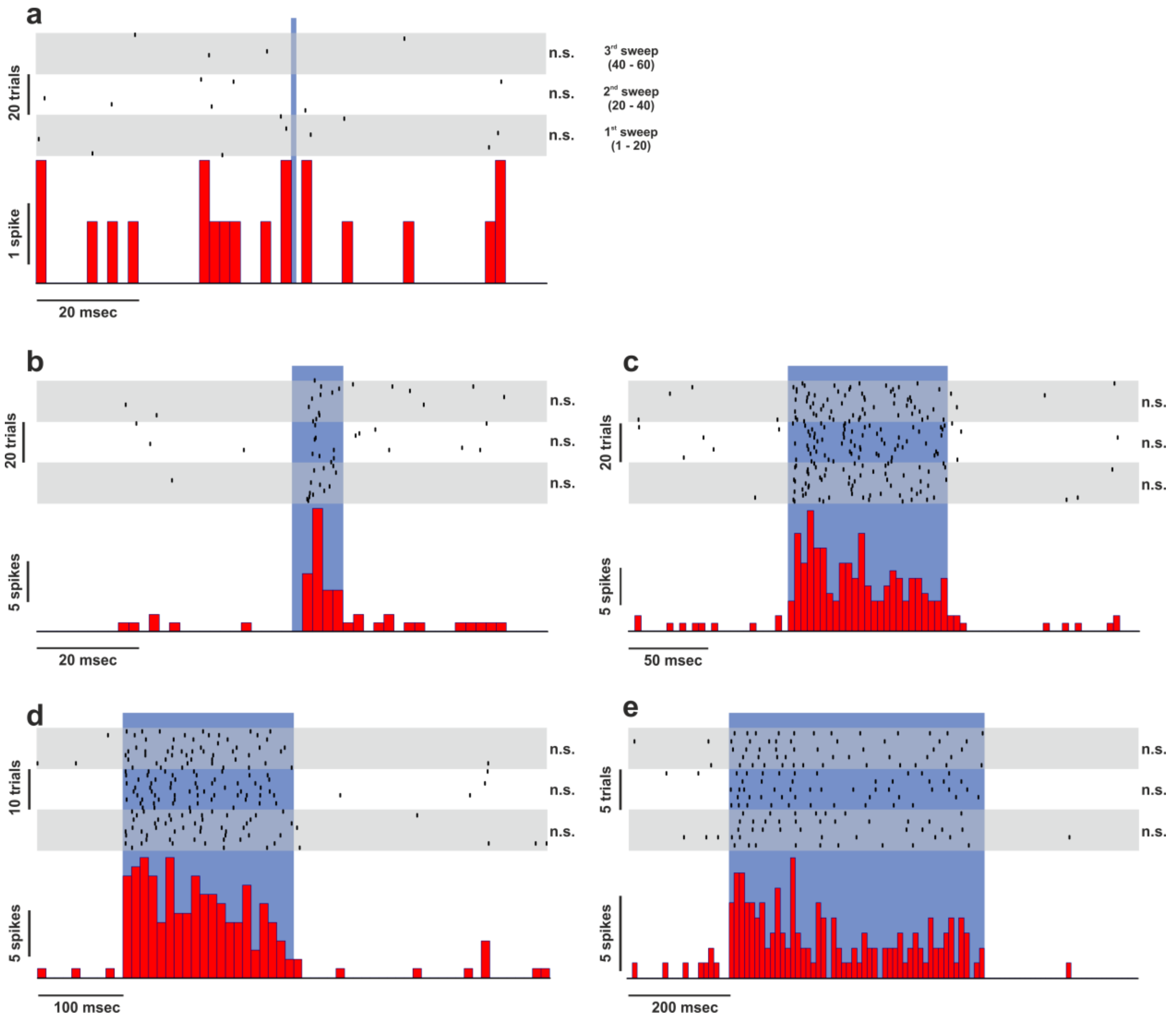

Supplementary Fig. 12. Single cell responses upon optical stimulation (Cell 2). (a) Raster plot (upper trace) and PSTH (lower trace) for the 1 ms stimulus duration (bin width: 2 ms). **(b)** Raster plot (upper trace) and PSTH (lower trace) for the 10 ms stimulus duration (bin width: 2 ms). **(c)** Raster plot (upper trace) and PSTH (lower trace) for the 100 ms stimulus duration (bin width: 4 ms). **(d)** Raster plot (upper trace) and PSTH (lower trace) for the 200 ms stimulus duration (bin width: 10 ms). **(e)** Raster plot (upper trace) and PSTH (lower trace) for the 500 ms stimulus duration (bin width: 10 ms).

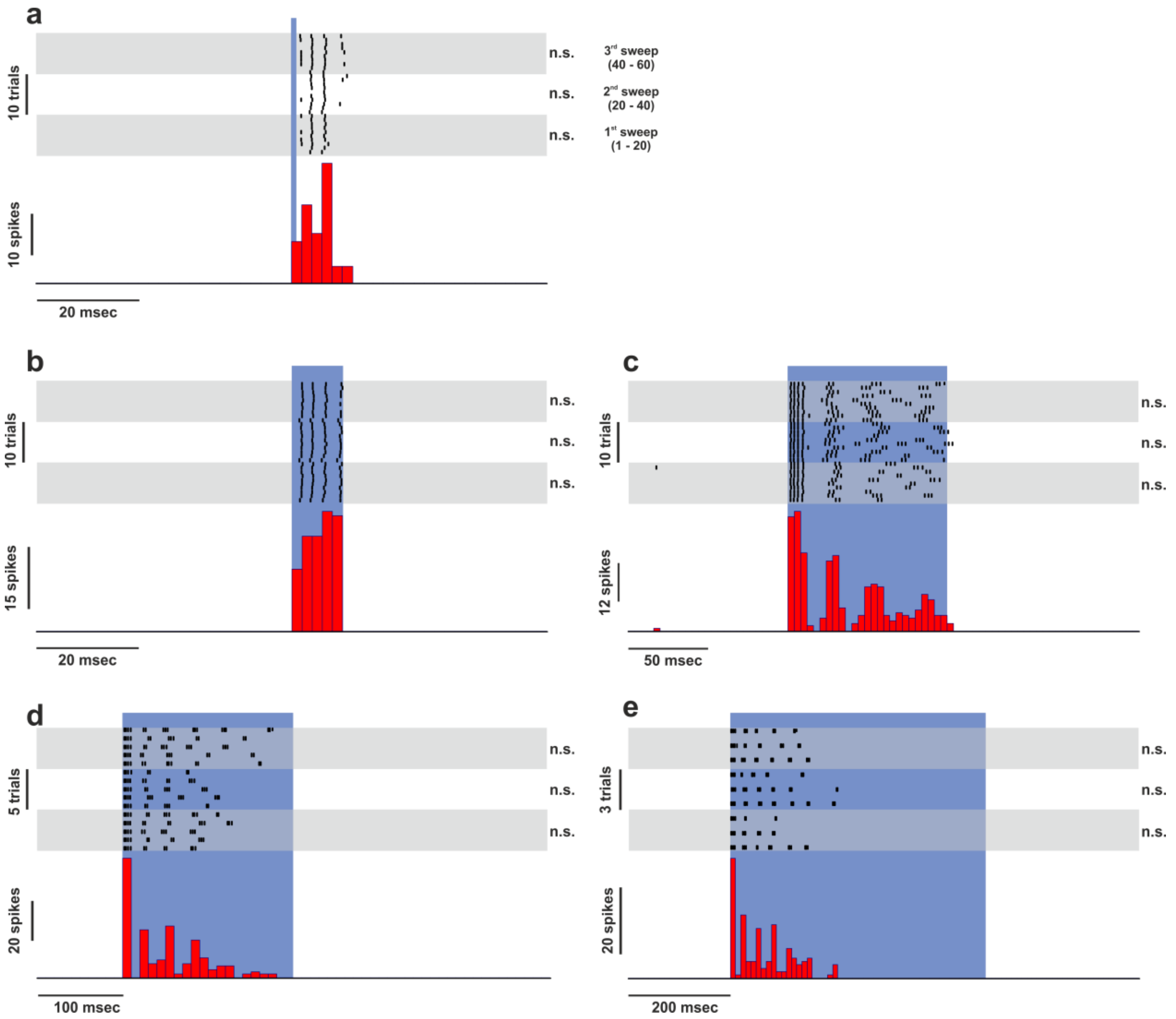

Supplementary Fig. 13. Single cell responses upon optical stimulation (Cell 5). (a) Raster plot (upper trace) and PSTH (lower trace) for the 1 ms stimulus duration (bin width: 2 ms). **(b)** Raster plot (upper trace) and PSTH (lower trace) for the 10 ms stimulus duration (bin width: 2 ms). **(c)** Raster plot (upper trace) and PSTH (lower trace) for the 100 ms stimulus duration (bin width: 4 ms). **(d)** Raster plot (upper trace) and PSTH (lower trace) for the 200 ms stimulus duration (bin width: 10 ms). **(e)** Raster plot (upper trace) and PSTH (lower trace) for the 500 ms stimulus duration (bin width: 10 ms).

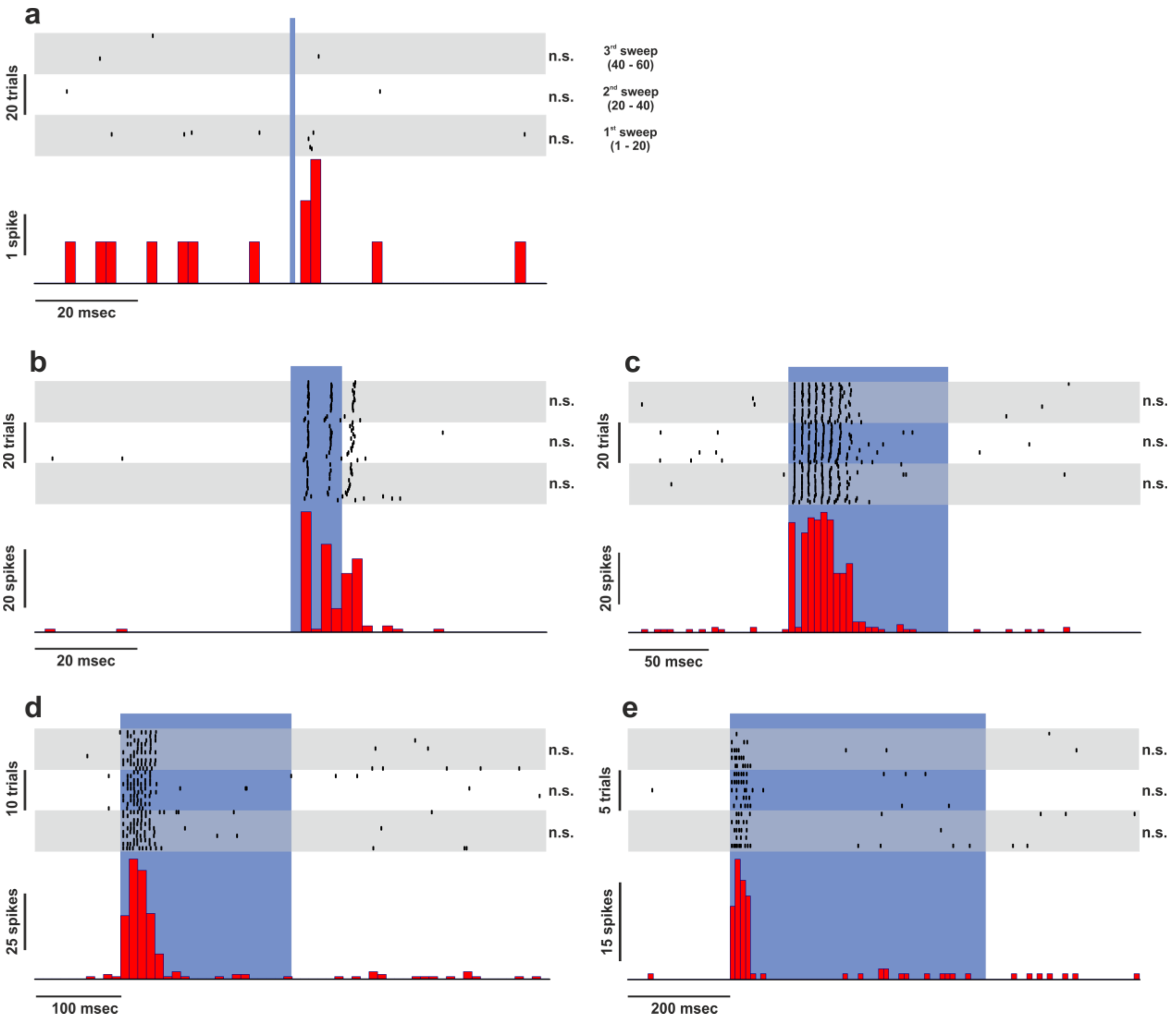

Supplementary Fig. 14. Single cell responses upon optical stimulation (Cell 6). (a) Raster plot (upper trace) and PSTH (lower trace) for the 1 ms stimulus duration (bin width: 2 ms). **(b)** Raster plot (upper trace) and PSTH (lower trace) for the 10 ms stimulus duration (bin width: 2 ms). **(c)** Raster plot (upper trace) and PSTH (lower trace) for the 100 ms stimulus duration (bin width: 4 ms). **(d)** Raster plot (upper trace) and PSTH (lower trace) for the 200 ms stimulus duration (bin width: 10 ms). **(e)** Raster plot (upper trace) and PSTH (lower trace) for the 500 ms stimulus duration (bin width: 10 ms).

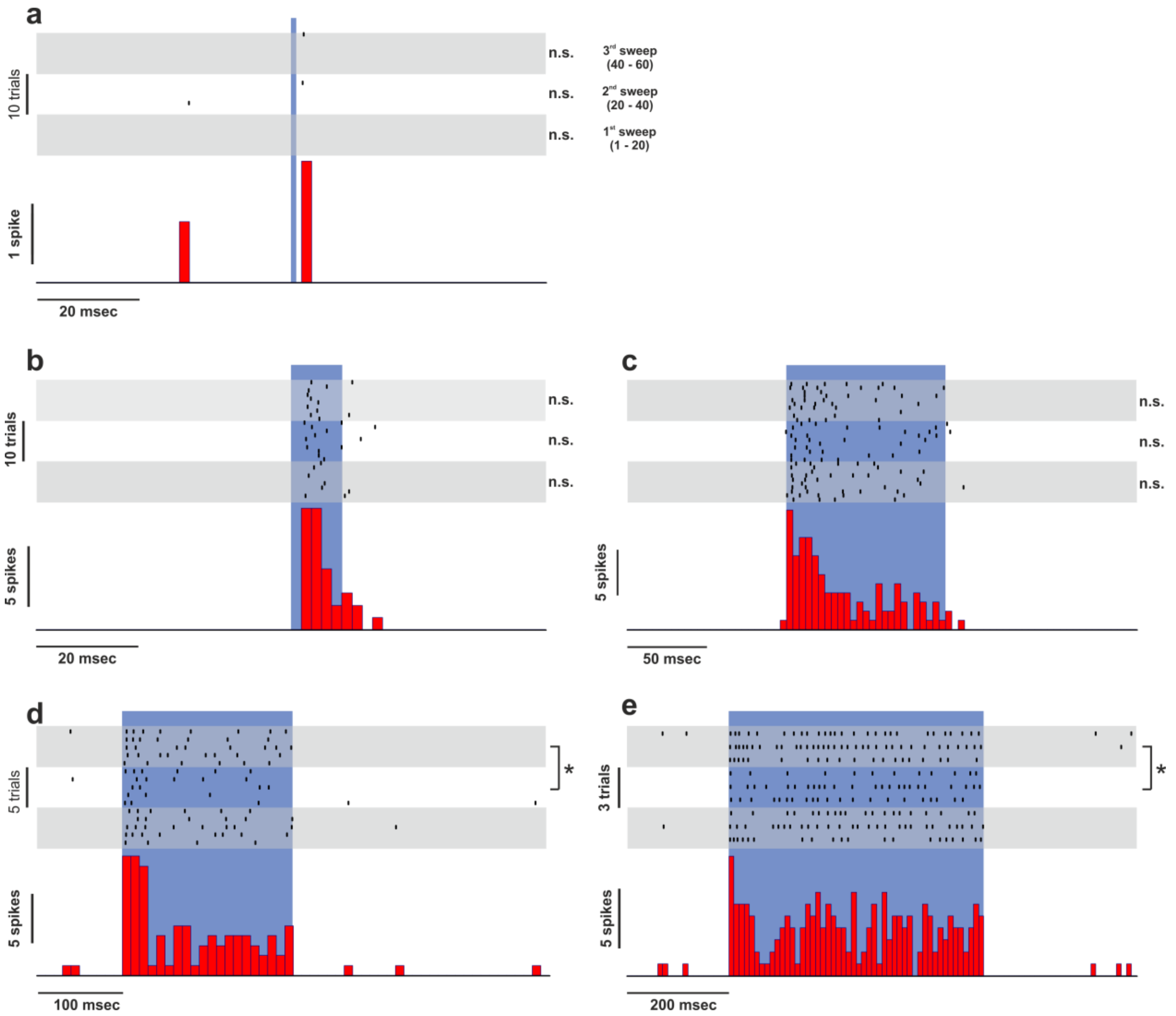

Supplementary Fig. 15. Single cell responses upon optical stimulation (Cell 7). (a) Raster plot (upper trace) and PSTH (lower trace) for the 1 ms stimulus duration (bin width: 2 ms). **(b)** Raster plot (upper trace) and PSTH (lower trace) for the 10 ms stimulus duration (bin width: 2 ms). **(c)** Raster plot (upper trace) and PSTH (lower trace) for the 100 ms stimulus duration (bin width: 4 ms). **(d)** Raster plot (upper trace) and PSTH (lower trace) for the 200 ms stimulus duration (bin width: 10 ms). **(e)** Raster plot (upper trace) and PSTH (lower trace) for the 500 ms stimulus duration (bin width: 10 ms).

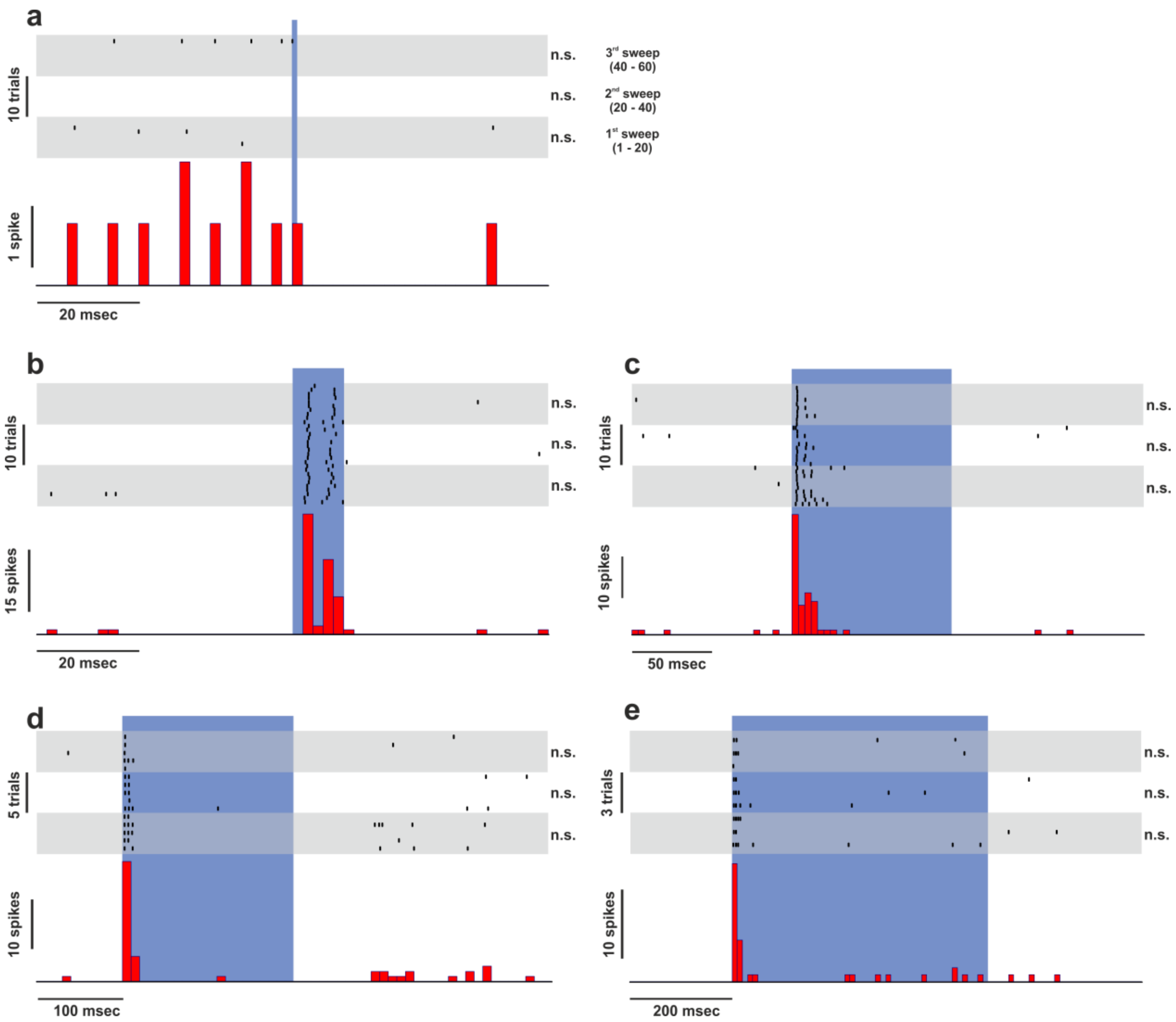

Supplementary Fig. 16. Single cell responses upon optical stimulation (Cell 8). (a) Raster plot (upper trace) and PSTH (lower trace) for the 1 ms stimulus duration (bin width: 2 ms). **(b)** Raster plot (upper trace) and PSTH (lower trace) for the 10 ms stimulus duration (bin width: 2 ms). **(c)** Raster plot (upper trace) and PSTH (lower trace) for the 100 ms stimulus duration (bin width: 4 ms). **(d)** Raster plot (upper trace) and PSTH (lower trace) for the 200 ms stimulus duration (bin width: 10 ms). **(e)** Raster plot (upper trace) and PSTH (lower trace) for the 500 ms stimulus duration (bin width: 10 ms).

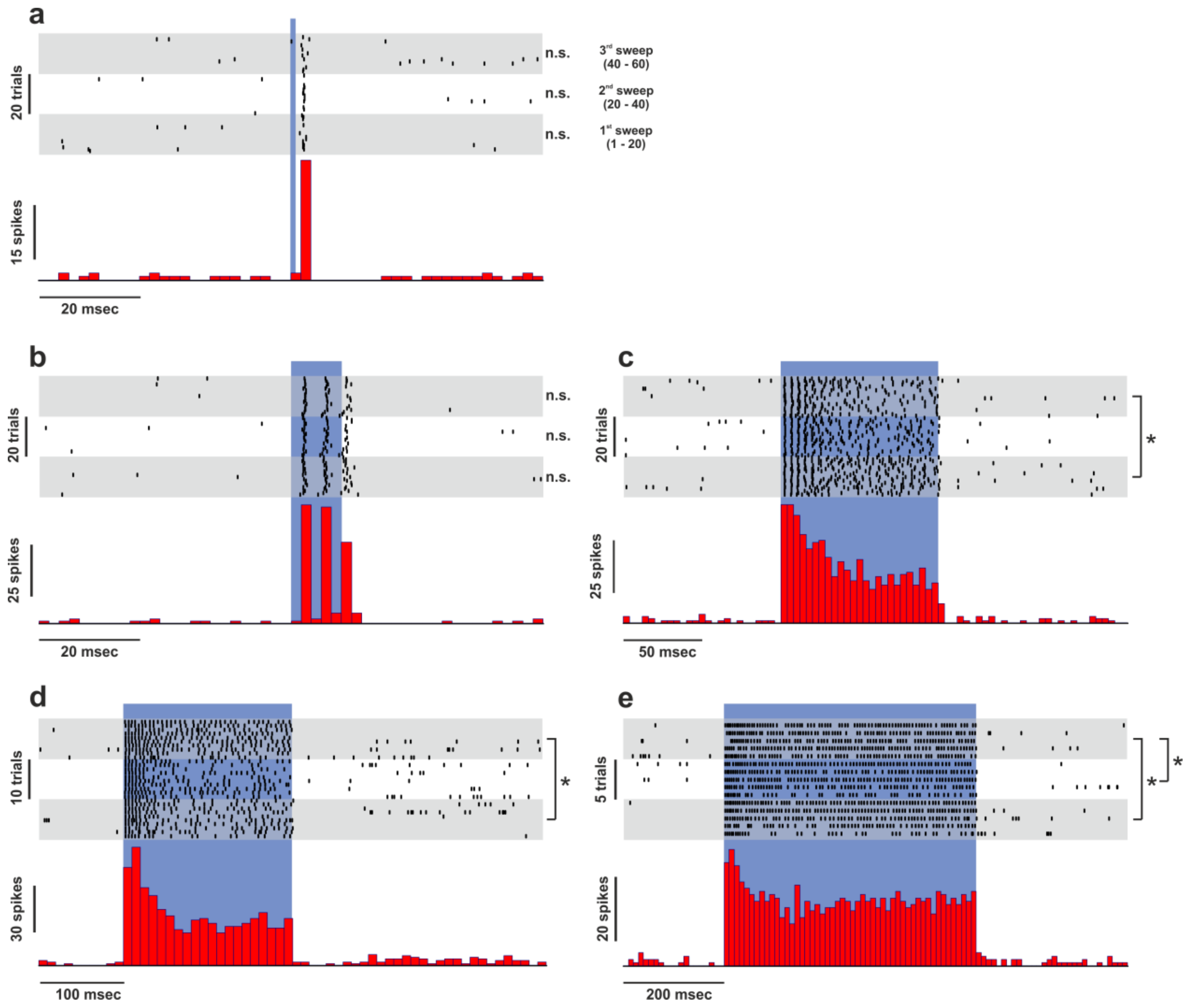

Supplementary Fig. 17. Single cell responses upon optical stimulation (Cell 9). (a) Raster plot (upper trace) and PSTH (lower trace) for the 1 ms stimulus duration (bin width: 2 ms). **(b)** Raster plot (upper trace) and PSTH (lower trace) for the 10 ms stimulus duration (bin width: 2 ms). **(c)** Raster plot (upper trace) and PSTH (lower trace) for the 100 ms stimulus duration (bin width: 4 ms). Please note the difference in the firing rates between the first and third sweep. The firing rates of the individual sweeps are given in Supplementary table 3. **(d)** Raster plot (upper trace) and PSTH (lower trace) for the 200 ms stimulus duration (bin width: 10 ms). Please note the difference in the firing rates between the first and third sweep. The firing rates of the individual sweeps are given in Supplementary table 3. **(e)** Raster plot (upper trace) and PSTH (lower trace) for the 500 ms stimulus duration (bin width: 10 ms). Please note the difference in the firing rates between the first and third sweep as well as the second and third sweep. The firing rates of the individual sweeps are given in Supplementary table 3.

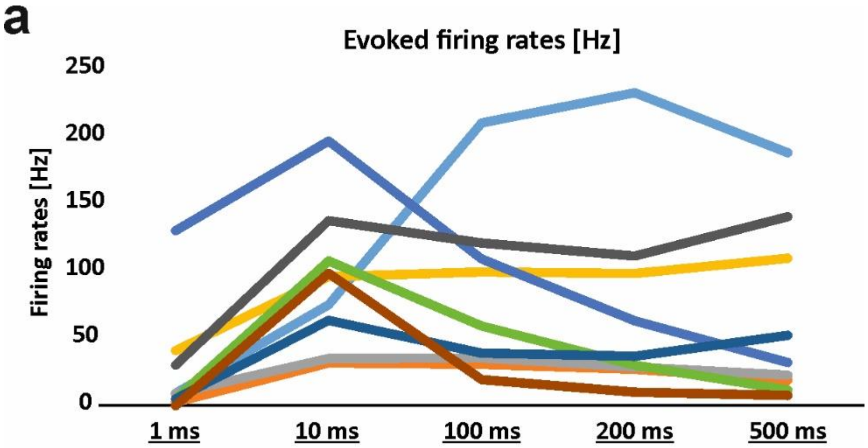

	1 ms	10 ms	100 ms	200 ms	500 ms
Cell 1	9.17	75	210	232.5	187.86
Cell 2	2.5	31.67	30.67	27	18.8
Cell 3	9.17	35	35.33	28.67	22.67
Cell 4	40.83	95.83	99.33	98.17	109.47
Cell 5	130	196.67	109	63	32
Cell 6	5	107.5	59.17	30	11.6
Cell 7	5	63.33	39	36.67	52.22
Cell 8	0	98.33	19.33	10	7.55
Cell 9	30	137.5	120.83	111.17	140.4

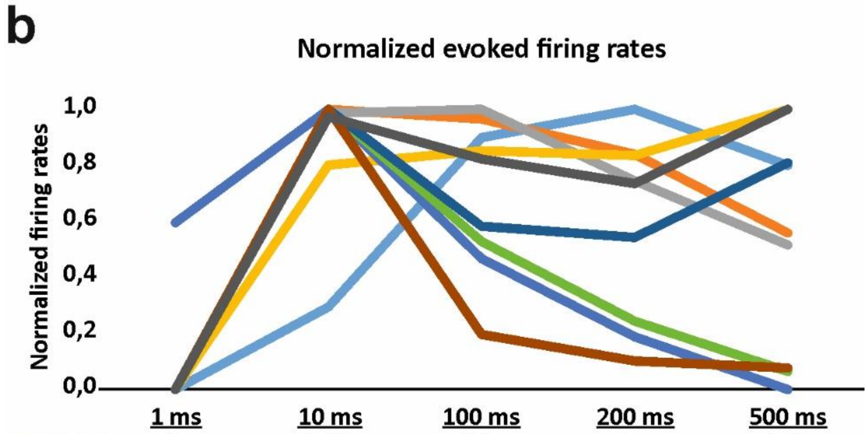

	1 ms	10 ms	100 ms	200 ms	500 ms
Cell 1	0	0.29	0.9	1	0.80
Cell 2	0	1	0.97	0.84	0.56
Cell 3	0	0.99	1	0.75	0.52
Cell 4	0	0.80	0.85	0.84	1
Cell 5	0.6	1	0.47	0.19	0
Cell 6	0	1	0.53	0.24	0.06
Cell 7	0	1	0.58	0.54	0.81
Cell 8	0	1	0.2	0.1	0.08
Cell 9	0	0.97	0.82	0.74	1

Supplementary Fig. 18. Absolute and relative evoked firing rates under light stimulation. (a) Average evoked firing rates during different stimulation durations. Please note that for the 1 ms and the 10 ms stimulation duration a 20 ms time window after stimulation onset was analyzed. In the table below the figure, values for each individual cell are given. **(b)** Normalized evoked firing rates.

Supplementary Table 2. Stimulus durations, inter pulse-interval (ms) and rate (Hz) for the two electrophysiological protocols (short and long)

Short protocol				Long protocol			
Stimulation duration [ms]	Inter-pulse-interval [ms]	Rate [Hz]	Repeats per sweep	Stimulation duration [ms]	Inter-pulse-interval [ms]	Rate [Hz]	Repeats per sweep
1	399	2.5	10	1	199	5	20
10	390	2.5	10	10	190	5	20
100	900	1	10	100	400	2	20
200	1800	0.5	5	200	800	1	10
500	1500	0.5	3	500	500	1	5

Supplementary Table 3. Summary of cell responses upon optical stimulation. For each cell, the spontaneous activity is given in the first block of the table. Following blocks summarize the cellular responses upon visual stimulation for each stimulus duration. Each sweep of stimulation is given separately. The labeling of the sweeps (gray/white background) corresponds to the figures in the single cell depiction. As a measure of variability, firing rates per bin of each individual sweep were statistically compared. If there were significant differences in the firing rate per bin between consecutive sweeps, values are printed in bold and marked with an asterisk. The last row of each block indicates if the optical stimulation resulted in a significant cell response compared to the non-stimulated periods within the time window of the respective Raster/PSTH time window. Please note that for the 1 ms and 10 ms stimulation duration a 20 ms time window after stimulation onset was analyzed.

		Cell 1	Cell 2	Cell 3	Cell 4	Cell 5	Cell 6	Cell 7	Cell 8	Cell 9
	**Spontaneous activity**	2.00	3.82	5.07	0	0	1.50	1.90	1.36	3.88
	**Standard deviation**	1.79	2.14	4.20	0	0	1.51	1.60	2.27	3.22
**1 ms stim. duration**	**Mean AP/bin sweep 1**	0.60	0.20	0.40	1.80	2.40	0.40	0	0	1.10
	**Standard deviation**	0.32	0.42	0.70	4.37	3.72	0.84	0	0	2.85
	**Mean AP/bin sweep 2**	0.40	0.10	0.40	1.50	2.30	0.10	0.10	0	1.30
	**Standard deviation**	0.70	0.32	0.70	4.40	3.37	0.32	0.32	0	3.77
	**Mean AP/bin sweep 3**	0.10	0	0.30	1.60	3.10	0.10	0.20	0	1.20
	**Standard deviation**	0.32	0	0.95	5.06	3.51	0.32	0.42	0	2.78
	**Mean AP/bin all sweeps**	1	0.3	1.1	4.9	7.8	0.6	0.3	0	3.6
	**Standard deviation**	1.56	0.67	1.73	13.78	9.83	1.08	0.67	0	9.34
	**Evoked firing rate [Hz]**	9.17	2.5	9.17	40.83	130	5	5	0	30
	**p-value**	0.14	0.66	0.38	**0.04**	**0.003**	0.55	0.54	------	0.35
**10 ms stim. duration**	**Mean AP/bin sweep 1**	2.90	1.40	1.60	4.10	4.00	3.90	1.20	2.10	5.60
	**Standard deviation**	3.00	1.96	2.32	6.92	4.40	4.95	1.32	3.73	7.49
	**Mean AP/bin sweep 2**	3.90	1.20	1.10	3.50	4.00	4.40	1.40	1.90	5.40
	**Standard deviation**	4.36	1.87	1.52	5.50	4.35	5.36	1.78	3.57	7.03
	**Mean AP/bin sweep 3**	2.20	1.20	1.50	3.90	3.80	4.60	1.20	1.90	5.50
	**Standard deviation**	2.70	1.87	1.90	5.72	4.73	6.24	1.69	3.18	7.89
	**Mean AP/bin all sweeps**	9	3.8	4.2	11.5	11.8	12.9	3.8	5.9	16.5
	**Standard deviation**	8.22	4.59	5.03	17.4	12.98	14.96	4.26	10.02	22.31
	**Evoked firing rate [Hz]**	75	31.67	35	95.83	196.67	107.5	63.33	98.33	137.5
	**p-value**	**< 0.001**	**0.01**	**0.02**	**0.003**	**0.007**	**< 0.001**	**0.005**	**0.02**	**0.003**
**100 ms stim. duration**	**Mean AP/bin sweep 1**	16.48	2.44	2.56	8.48	3.68	4.72	1.68	1.08	**11.48 ***
	**Standard deviation**	2.82	1.53	1.56	3.57	4.53	6.33	1.46	2.41	3.94
	**Mean AP/bin sweep 2**	17.08	2.48	3.04	7.72	4.60	5.05	1.40	0.68	9.00
	**Standard deviation**	3.00	1.69	1.62	2.97	4.32	6.60	1.38	2.04	4.44
	**Mean AP/bin sweep 3**	16.84	2.44	2.88	7.64	4.80	4.44	1.60	0.56	**8.52 ***
	**Standard deviation**	3.94	1.56	2.05	3.47	4.80	6.16	1.47	2.06	5.16
	**Mean AP/bin all sweeps**	50.4	7.36	8.48	23.84	13.08	14.2	4.68	2.28	29
	**Standard deviation**	6.68	3.34	3.47	8.47	12.81	18.57	3.64	5.83	12.21
	**Evoked firing rate [Hz]**	210	30.67	35.33	99.33	109	59.17	39	19.33	120.83
	**p-value**	**< 0.001**	**< 0.001**	**< 0.001**	**< 0.001**	**< 0.001**	**< 0.001**	**< 0.001**	**< 0.007**	**< 0.001**
**200 ms stim. duration**	**Mean AP/bin sweep 1**	23.35	2.50	2.85	10.40	3.10	3.50	1.90	0.60	**10.10 ***
	**Standard deviation**	3.07	1.54	1.81	1.96	4.99	6.19	1.59	2.09	5.47
	**Mean AP/bin sweep 2**	23.25	3.00	2.95	9.85	2.75	2.80	**1.25 ***	0.55	10.00
	**Standard deviation**	4.06	1.97	1.39	2.25	4.64	5.47	1.65	2.01	6.15
	**Mean AP/bin sweep 3**	23.15	2.60	2.80	9.20	3.60	2.70	**2.35 ***	0.35	**13.25 ***
	**Standard deviation**	3.34	1.05	1.74	2.46	4.98	5.29	1.23	1.35	3.80
	**Mean AP/bin all sweeps**	69.75	8.1	8.6	29.5	9.45	9	5.5	1.5	33.35
	**Standard deviation**	8.75	3.19	3.78	6.02	14.18	16.64	3.83	5.41	14.34
	**Evoked firing rate [Hz]**	232.5	27	28.67	98.17	63	30	36.67	10	111.17
	**p-value**	**< 0.001**	**< 0.001**	**< 0.001**	**< 0.001**	**< 0.001**	**0.046**	**< 0.001**	0.94	**< 0.001**
**500 ms stim. duration**	**Mean AP/bin sweep 1**	10.04	0.82	1.14	5.74	0.78	0.64	1.54	0.26	**6.25 ***
	**Standard deviation**	3.75	0.83	1.05	1.07	2.13	1.89	0.91	0.96	2.00
	**Mean AP/bin sweep 2**	9.16	0.98	1.08	5.40	1.10	0.66	**1.26 ***	0.24	**6.65 ***
	**Standard deviation**	4.06	1.00	0.78	1.39	2.26	1.80	0.85	0.92	1.91
	**Mean AP/bin sweep 3**	8.98	1.02	1.18	5.28	1.00	0.44	**1.90 ***	0.18	**7.75 ***
	**Standard deviation**	3.88	0.98	0.90	1.21	2.20	1.43	1.11	0.75	2.21
	**Mean AP/bin all sweeps**	28.18	2.82	3.4	16.42	2.88	1.74	4.7	0.68	21.04
	**Standard deviation**	10.88	1.84	1.8	2.67	6.43	4.97	1.97	2.54	4.22
	**Evoked firing rate [Hz]**	187.86	18.8	22.67	109.47	32	11.6	52.22	7.55	140.4
	**p-value**	**< 0.001**	**< 0.001**	**< 0.001**	**< 0.001**	**< 0.001**	**0.01**	**< 0.001**	**0.005**	**< 0.001**

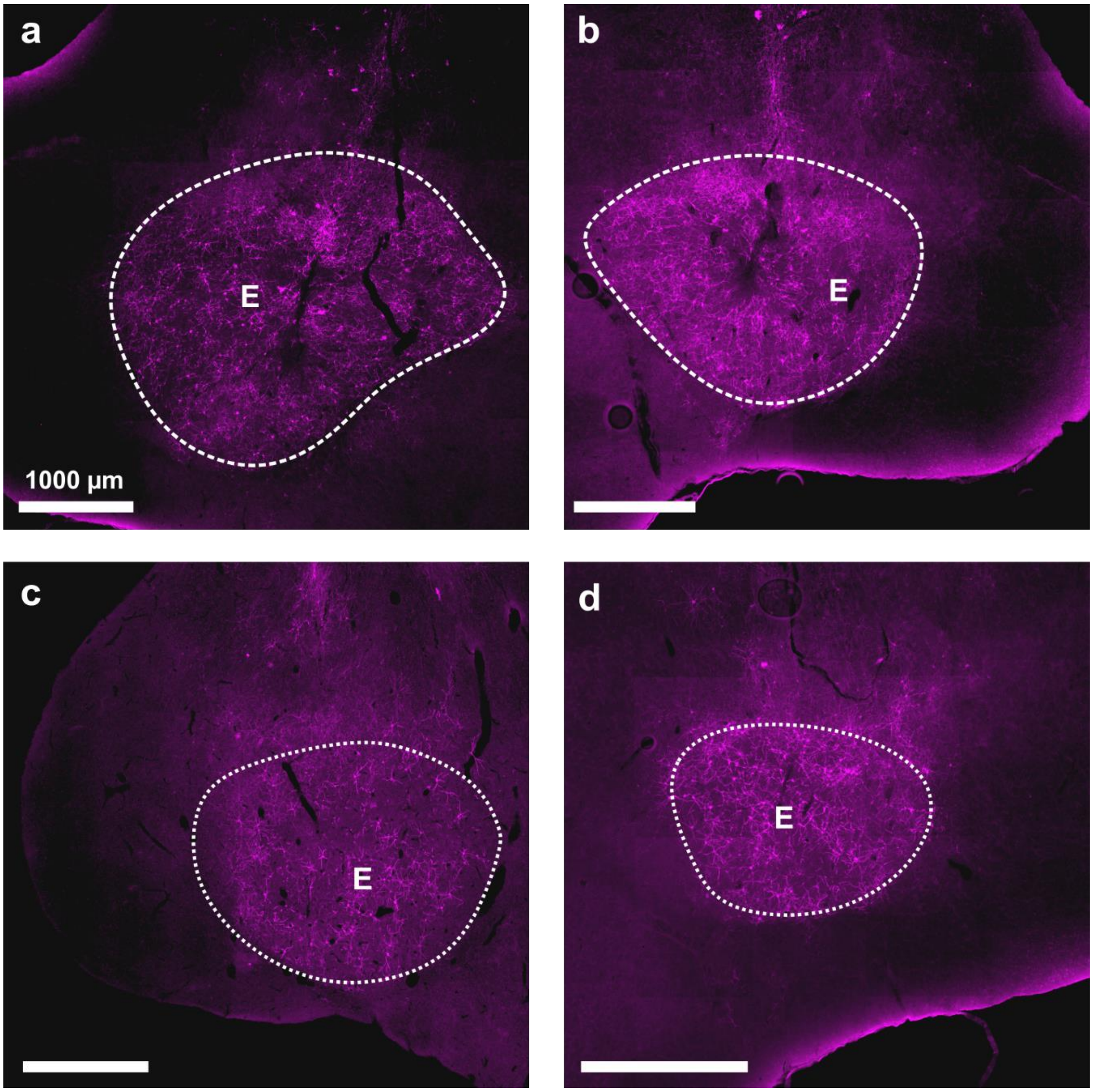

Supplementary Fig. 19. ChR2 expression in the entopallium of the four recorded hemispheres. All scale bars represent 1000 µm. The entopallium is highlighted.

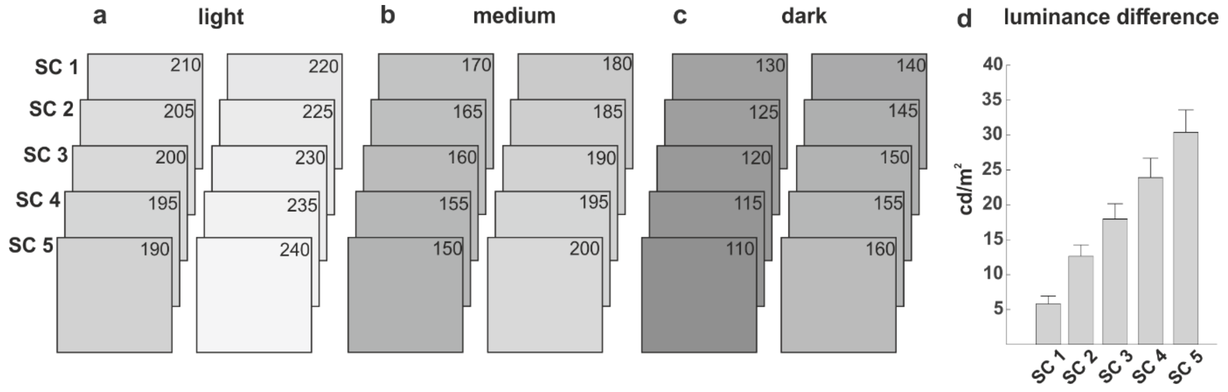

Supplementary Fig. 20. Stimuli of the behavioral paradigm. (a) Stimuli of the five different stimulus classes (SC) with high RGB values (mean RGB value 215). **(b)** Stimuli of the five different SC with medium RGB values (mean RGB value 175). **(c)** Stimuli of the five different SC with low RGB values (mean RGB value 135). The RGB value difference between the two grey scale stimuli in SC1 was 10 (mean luminance difference 5.81 cd/m^2), in SC2 20 (mean luminance difference 12.56 cd/m^2), in SC3 30 (mean luminance difference 17.96 cd/m^2), in SC4 40 (mean luminance difference 23.91 cd/m^2) and in SC5 50 (mean luminance difference 30.39 cd/m^2). All RGB values are written in the upper right corner in all images. **(d)** Luminance differences were measured with a photometer. Mean luminance difference gradually increased from SC1 to SC5. The error bars represent the standard error of the mean.

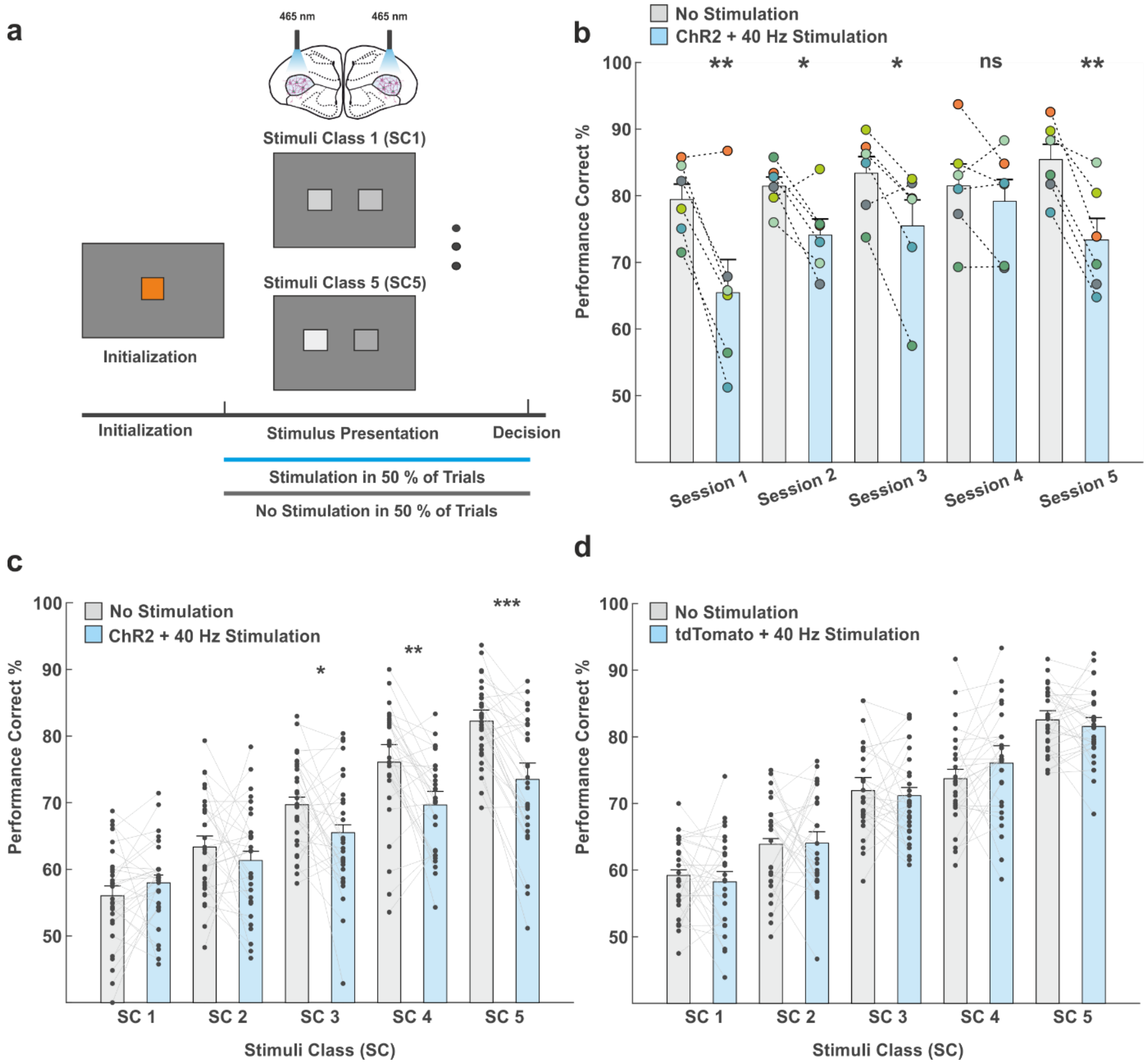

Supplementary Fig. 21. Transient 40 Hz activation of ChR2 expressing cells in the avian entopallium reduces contrast sensitivity. (a) Schematic illustration of the experimental procedure. Pigeons were conditioned to discriminate grey-scales of different stimulus classes (SC). SC1 consisted of grey-scale pictures that were difficult to discriminate and SC5 consisted of grey-scale pictures that were easy to discriminate. Pigeons were stimulated in half of the trials in a given session and stimulation took place during the whole stimulus presentation phase or until the animal responded. **(b)** Depicts the 40 Hz stimulation effect on the performance in SC5 over all five sessions for the ChR2 group (n = 6). Each data point represents the performance in one session of one pigeon. Stimulated and unstimulated performances within each session/pigeon have been connected with lines. Colors represent performances of individual animals (session 1 SC5 ($t_{(5)}$ = 4.126, p = .009), session 2 SC5 ($t_{(5)}$ = 2.832, p = .037), session 3 SC5 ($t_{(5)}$ = 2.935, p = .032), session 4 SC5 ($t_{(5)}$ = 1.029, p = .351), session 5 SC5 ($t_{(5)}$ = 5.616, p = .002). However, even though session 4 SC5 was not significant, in this session a significant effect was seen in SC4 ($t_{(5)}$ = 2.661, p = .045). **(c)** Depicts the 40 Hz stimulation effect on the performance of the ChR2 group (n = 6, the same data is shown in main text Fig. 7 c). To visualize performances of individual sessions/pigeons, the raw data of all sessions/pigeons is plotted. Furthermore, stimulated and unstimulated performances within each session/pigeon have been connected with lines. **(d)** Depicts the 40 Hz stimulation effect on the performance of the tdTomato group (n = 6, the same data is shown in main text Fig. 7 d). To visualize performances of individual sessions/pigeons, the raw data of all sessions/pigeons is plotted. Furthermore, stimulated and unstimulated performances within each session/pigeon have been connected with lines. Error bars represent the standard error of the mean (SEM). ***p < .001, **p < .01, *p < .05

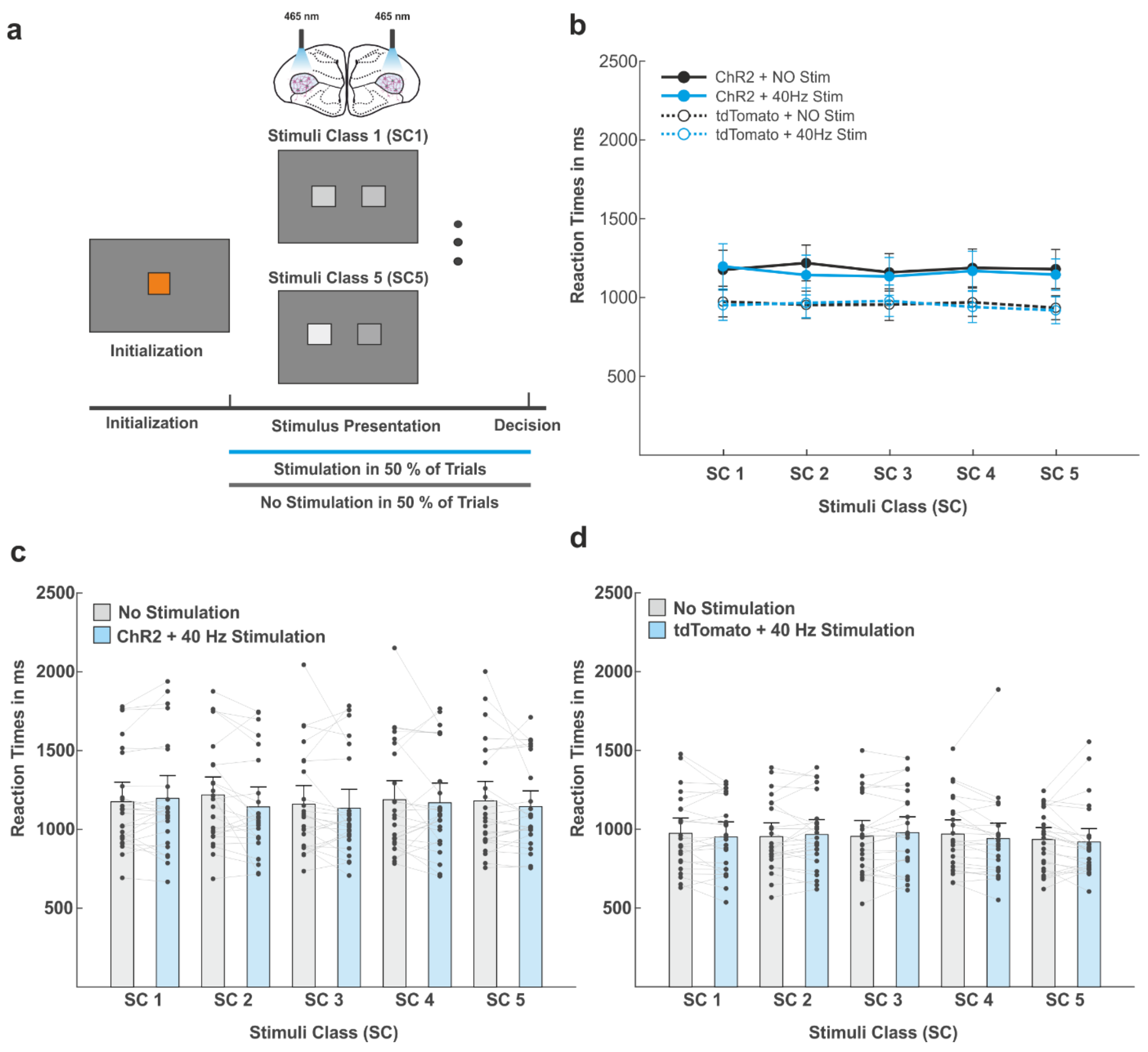

Supplementary Fig. 22. Transient 40 Hz activation of ChR2 expressing cells in the avian entopallium does not influence reaction times. (a) Schematic illustration of the experimental procedure. **(b)** There is no difference between reaction times in stimulated and unstimulated trials. The control group (n = 6) was a bit faster than the experimental group (n = 6). The reaction time difference between the experimental and control groups did not reach significance ($F_{(1,10)} = 2.067$, $p = .181$, $\eta_p^2 = .171$) **(c)** Depicts the 40 Hz stimulation effect on the reaction times of the ChR2 group (the same data is shown in main text Fig. 7 e). To visualize performances of individual sessions/pigeons, the raw data of all sessions/pigeons is plotted. Furthermore, stimulated and unstimulated performances within each session/pigeon have been connected with lines. **(d)** Depicts the 40 Hz stimulation effect on the reaction times of the tdTomato group (the same data is shown in main text Fig. 7 f). To visualize performances of individual sessions/pigeons, the raw data of all sessions/pigeons is plotted. Furthermore, stimulated and unstimulated performances within each session/pigeon have been connected with lines. Error bars represent the standard error of the mean (SEM).

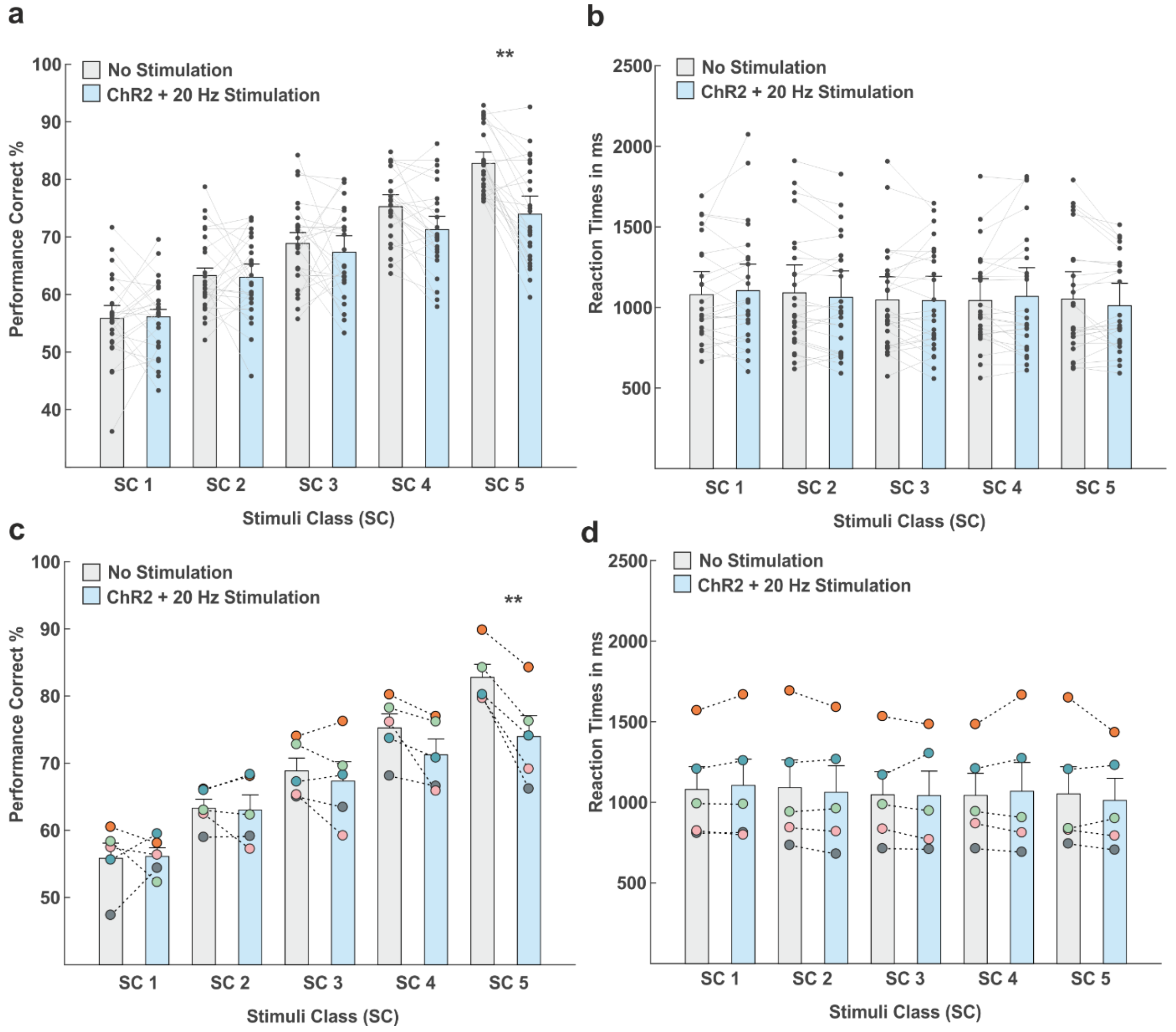

Supplementary Fig. 23. Transient 20 Hz activation of ChR2 expressing cells in the avian entopallium reduces contrast sensitivity in SC 5. (a) Depicts the 20 Hz stimulation effect on the performance for the ChR2 group (n = 5). Each data point represents the performance in one session of one pigeon. Stimulated and unstimulated performances within each session/pigeon have been connected with lines**.** There was no main effect of session ($F_{(4,16)}$ = .469, p = .757, η_p^2= .105), 20 Hz stimulation ($F_{(1,4)}$ = 6.666, p = .061, η_p^2= .625) and stimulus classes ($F_{(4,16)}$ = 72.059, p < .001, η_p^2= .947) on the accuracy of grey-scale visual discrimination. However, there was an interaction between "20 Hz stimulation" and "stimulus classes" ($F_{(4,16)}$ = 6.810, p = .002, η_p^2= .630). Bonferroni corrected pairwise comparisons revealed that optogenetic stimulation significantly impaired the performance for stimuli of SC5. **(b)** Depicts the 20 Hz stimulation effect on the reaction times for the ChR2 group (n = 5). Each data point represents the performance in one session of one pigeon. Stimulated and unstimulated performances within each session/animal have been connected with lines**.** There was no main effect of session ($F_{(4,16)}$ = 1.167, p = .362, η_p^2= .226), 20 Hz stimulation ($F_{(1,4)}$ = .063, p = .814, η_p^2= .016) and stimulus classes ($F_{(4,16)}$ = 2.533, p = .081, η_p^2= .388) on the reaction times. Furthermore, there was no interaction between "20 Hz stimulation" and "stimulus classes" ($F_{(4,16)}$ = .717, p = .593, η_p^2= .152). **(c)** Depicts the same data as (a) but the session means are plotted for every pigeon in individual colors. **(d)** Depicts the same data as in (b), but the session means are plotted for every pigeon in individual colors. Error bars represent the standard error of the mean (SEM). **p < .01. Dots represent raw data of all 5 pigeons tested in 5 sessions.

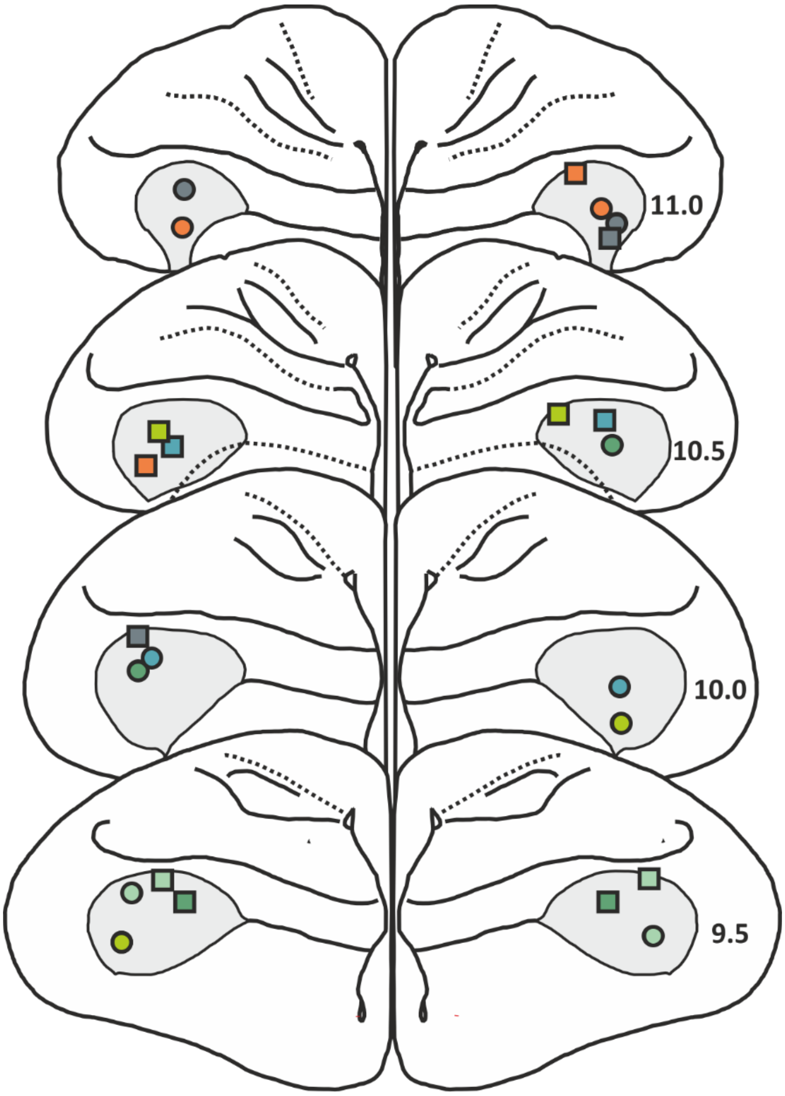

Supplementary Fig. 24. Schematic illustration of the cannula tips within the entopallium. Dots represent the cannula tips of the experimental group, squares represent cannula tips of the control group.

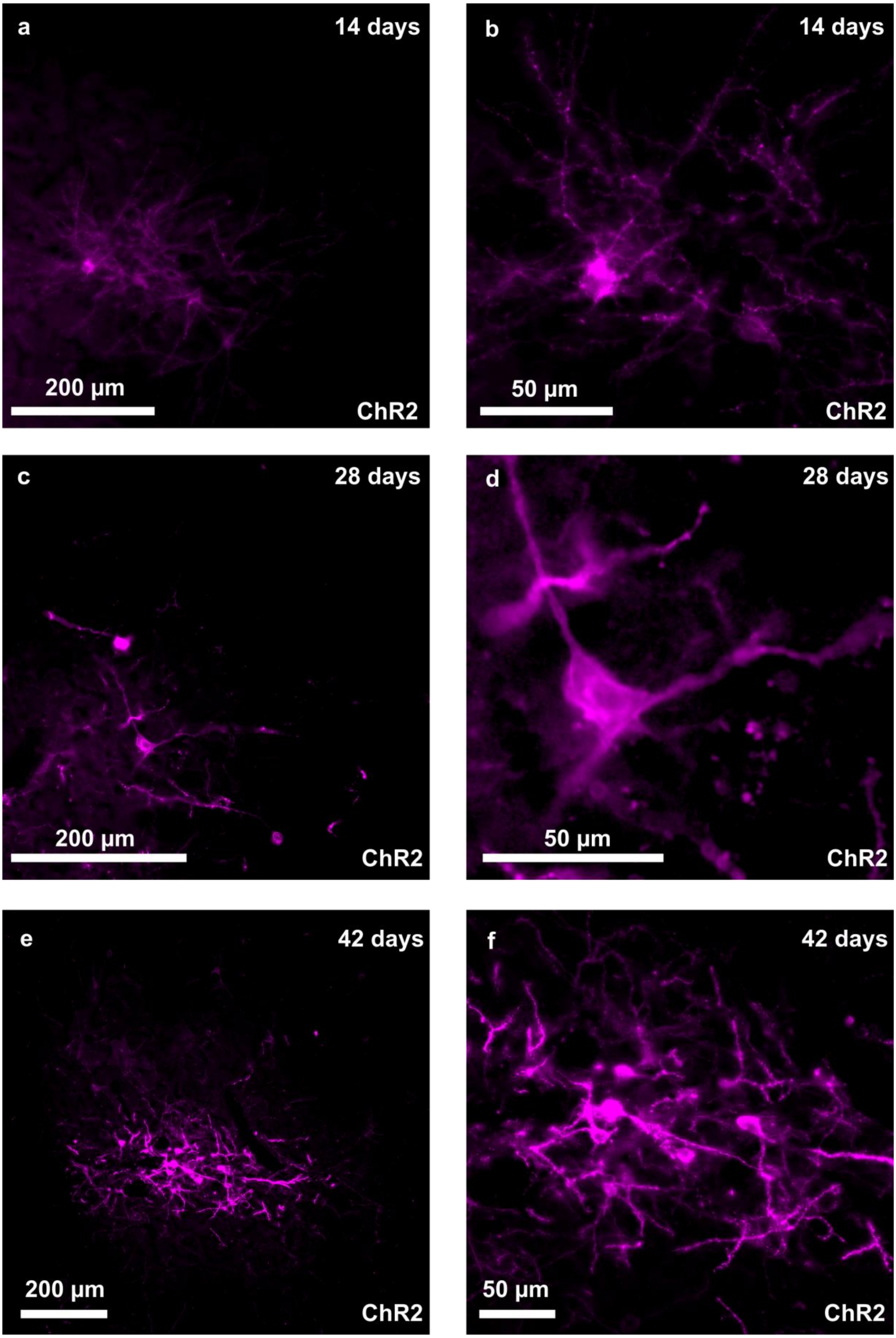

Supplementary Fig. 25. ChR2 expression increases over time. (a, b) ChR2 expression 14 days after AAV1-hSyn-ChR2 injections. **(c, d)** ChR2 expression 28 days after AAV1-hSyn-ChR2 injections. **(e, f)** ChR2 expression 42 days after AAV1-hSyn-ChR2 injections. Scale bars are specified within the images.

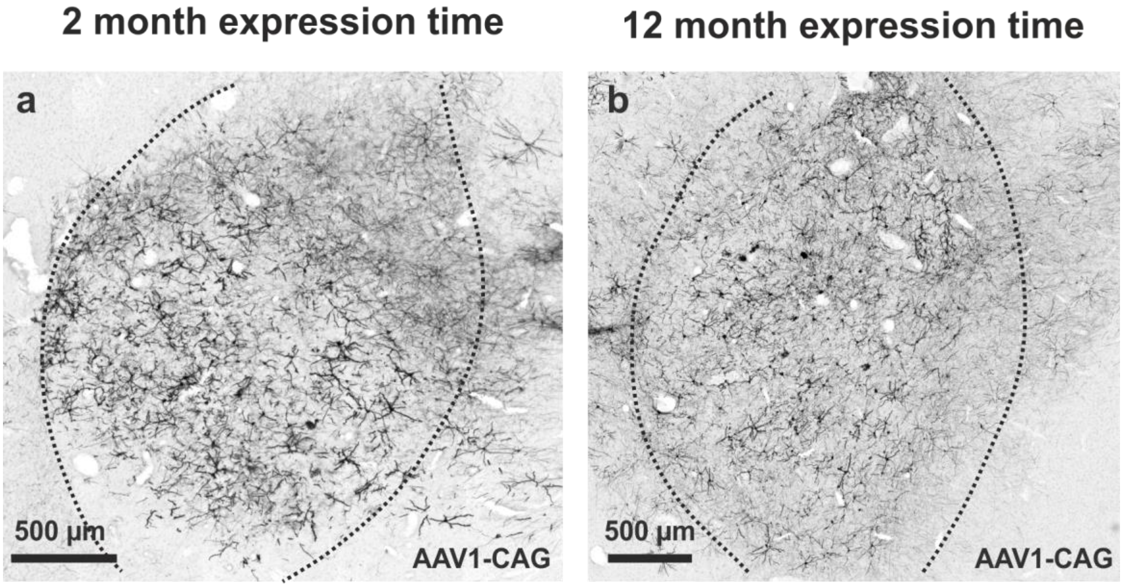

Supplementary Fig. 26. AAV1-CAG-ChR2 leads to stable long-term ChR2 expression up to one year. (a) ChR2 expression 2 months after AAV1-CAG-ChR2 injections counterstained against ChR2 with a DAB staining. **(b)** ChR2 expression one year after AAV1-CAG-ChR2 injections counterstained against ChR2 with a 3,3' diaminobenzidine (DAB) reaction staining. Scale bars represent 500 µm.
