# Supplementary material for: AAV1 is the optimal viral vector for optogenetic experiments in pigeons (Columba livia)
Source: Commun Biol. 2021 Jan 22;4:100. doi: 10.1038/s42003-020-01595-9 (PMC7822860; doi:10.1038/s42003-020-01595-9)
Supplement: Supplementary file 3 — Description of Supplementary Files [file 42003_2020_1595_MOESM3_ESM.pdf]

Description of Additional Supplementary Files

File Name: Supplementary Data 1

Description: Source data underlying the main text figures.
