# Supplementary material for: AAV1 is the optimal viral vector for optogenetic experiments in pigeons (Columba livia)
Source: Commun Biol. 2021 Jan 22;4:100. doi: 10.1038/s42003-020-01595-9 (PMC7822860; doi:10.1038/s42003-020-01595-9)
Supplement: Supplementary file 5 — Reporting Summary [file 42003_2020_1595_MOESM5_ESM.pdf]

nature research

Corresponding author(s): Noemi Rook

Last updated by author(s): Nov 12, 2020

Reporting Summary

Nature Research wishes to improve the reproducibility of the work that we publish. This form provides structure for consistency and transparency in reporting. For further information on Nature Research policies, see our Editorial Policies and the Editorial Policy Checklist.

Statistics

For all statistical analyses, confirm that the following items are present in the figure legend, table legend, main text, or Methods section.

n/a	Confirmed	
☐	☒	The exact sample size (n) for each experimental group/condition, given as a discrete number and unit of measurement
☐	☒	A statement on whether measurements were taken from distinct samples or whether the same sample was measured repeatedly
☐	☒	The statistical test(s) used AND whether they are one- or two-sided<br>*Only common tests should be described solely by name; describe more complex techniques in the Methods section.*
☒	☐	A description of all covariates tested
☐	☒	A description of any assumptions or corrections, such as tests of normality and adjustment for multiple comparisons
☐	☒	A full description of the statistical parameters including central tendency (e.g. means) or other basic estimates (e.g. regression coefficient) AND variation (e.g. standard deviation) or associated estimates of uncertainty (e.g. confidence intervals)
☐	☒	For null hypothesis testing, the test statistic (e.g. F, t, r) with confidence intervals, effect sizes, degrees of freedom and P value noted<br>*Give P values as exact values whenever suitable.*
☒	☐	For Bayesian analysis, information on the choice of priors and Markov chain Monte Carlo settings
☒	☐	For hierarchical and complex designs, identification of the appropriate level for tests and full reporting of outcomes
☐	☒	Estimates of effect sizes (e.g. Cohen's d, Pearson's r), indicating how they were calculated

Our web collection on statistics for biologists contains articles on many of the points above.

Software and code

Policy information about availability of computer code

Data collection	Image acquisition was performed with Zeiss Axio Imager M1 microscope and the software ZEN 2.3 blue edition (Carl Zeiss MicroImaging, Göttingen, Germany). The behavioral paradigm was written in MATLAB R2017a using the Biopsychology Toolbox (available at: http://biopsytoolbox.sourceforge.net/). Custom paradigm codes will be made available upon request.
Data analysis	The analysis of viral vector efficiencies was performed with ZEN 2.3 pro image analysis tool wizard and ZEN 2.3 lite (Carl Zeiss MicroImaging, Göttingen, Germany). The behavioral analysis codes were written in MATLAB R2017a using Biopsychology Toolbox (available at: http://biopsytoolbox.sourceforge.net/). Custom analysis codes will be made available upon request.

For manuscripts utilizing custom algorithms or software that are central to the research but not yet described in published literature, software must be made available to editors and reviewers. We strongly encourage code deposition in a community repository (e.g. GitHub). See the Nature Research guidelines for submitting code & software for further information.

Data

Policy information about availability of data

All manuscripts must include a data availability statement. This statement should provide the following information, where applicable:

- Accession codes, unique identifiers, or web links for publicly available datasets
- A list of figures that have associated raw data
- A description of any restrictions on data availability

The data that support the findings of this study are included in the Article and its Supplementary Information or available from the corresponding author upon request. The source data of the main text figures is available in Supplementary Data 1.

Field-specific reporting

Please select the one below that is the best fit for your research. If you are not sure, read the appropriate sections before making your selection.

- [x] Life sciences
- [ ] Behavioural & social sciences
- [ ] Ecological, evolutionary & environmental sciences

For a reference copy of the document with all sections, see nature.com/documents/nr-reporting-summary-flat.pdf

Life sciences study design

All studies must disclose on these points even when the disclosure is negative.

Sample size	For the viral transfection study, sample size was determined based on previously published viral transfection studies that have been published in other species such as mice (Taymans et al., 2007), rats (Burger et al., 2004), primates (Markakis et al.,2010) and cats (Vite et al.,2003). For all viral vectors, a minimum of five injections were performed in separate hemispheres of at least three pigeons.For the behavioral analysis, sample size was determined on previously published entopallium lesion studies that investigated visual discrimination in pigeons (Nguyen et al., 2004; Cook et al., 2013). Six control and six experimental pigeons were tested in five sessions. Furthermore, we calculated the effect sizes for our main findings. The obtained effects of our study were all large according to Cohen (1988). Therefore, we are convinced that the obtained results are robust and reliable.
Data exclusions	For the behavioral experiments, pigeons were tested in sessions with 600 trials in total that contained stimulated and unstimulated trials of all five stimulus classes. Only sessions where the pigeons engaged in at least 50 % of trials were considered for the further analysis to make sure that pigeons were paying attention and that a reliable amount of trials was gathered per condition. Only sessions where the pigeons reached a criterion of 75% correct responses in at least one stimulus class were considered for further analysis to make sure that pigeons had successfully learnt the discrimination as their performance was significantly above chance level.For AAV9-hSyn only qualitative findings are presented. This serotype was not included in the statistical analysis as it did not result in ChR2 expression. Apart from that, no data was excluded.
Replication	All stainings were performed twice with separate brain series to qualitatively confirm the reproducibility of stainings .For the behavioral study, six pigeons were retested in five sessions to show that the effect of stimulation is reliable, robust and can be replicated. We could not find a main effect of session ($F(4,40) = .802$, $p = .531$, $\eta p2= .074$) nor an interaction of stimulation and session ($F(4,40) = .626$, $p = .647$, $\eta p2= .059$) indicating that optical stimulation had similar effects in all tested sessions. Furthermore, the stimulation effect in SC5 was present in 4 out of 5 sessions (session 1 SC5 ($t(5) = 4.126$, $p = .009$), session 2 SC5 ($t(5) = 2.832$, $p = .037$), session 3 SC5 ($t(5) = 2.935$, $p = .032$), session 4 SC5 ($t(5) = 1.029$, $p = .351$), session 5 SC5 ($t(5) = 5.616$, $p = .002$). However, even though in session 4 SC5 was not significant, in this session a significant effect was seen in SC4 ($t(5) = 2.661$, $p = .045$).
Randomization	Pigeons were assigned to the experimental and control groups in a randomized order.
Blinding	The quantification of ChR2 expressing cells and the measurement of ChR2 expressing area was performed blinded. The experimenter was not aware of the serotype they investigated. Furthermore, the behavioral performance of both experimental and control pigeons was recorded in MATLAB and was thus not subject to observation (blinded). Furthermore, the experimenter that trained and tested the pigeons was not aware of the group allocation.

Reporting for specific materials, systems and methods

We require information from authors about some types of materials, experimental systems and methods used in many studies. Here, indicate whether each material, system or method listed is relevant to your study. If you are not sure if a list item applies to your research, read the appropriate section before selecting a response.

Materials & experimental systems

n/a	Involved in the study
[ ]	[x] Antibodies
[x]	[ ] Eukaryotic cell lines
[x]	[ ] Palaeontology and archaeology
[ ]	[x] Animals and other organisms
[x]	[ ] Human research participants
[x]	[ ] Clinical data
[x]	[ ] Dual use research of concern

Methods

n/a	Involved in the study
[x]	[ ] ChIP-seq
[x]	[ ] Flow cytometry
[x]	[ ] MRI-based neuroimaging

Antibodies

Antibodies used	Antibodies including their supplier name are all specified at the specific location in method section of the manuscript
Validation	Primary antibody specificity was either evaluated with western blots, based on previously published literature or investigated with immunhistochemical stainings in negative control samples. For anti-ChR2, brain sections with injections of AAV1-CAG-ChR2, AAV1-

CAG-tdTomato and sections without injections were stained with the same protocol in the same pass. Only in the brain sections where AAV1-CAG-ChR2 was injected a signal occurred. The specificity of the c-Fos antibody (Santa Cruz, sc-253) has previously been investigated in a different study using pigeons (Nimpf et al., 2019). For anti-NeuN, western Blots have been performed.

Animals and other organisms

Policy information about studies involving animals; ARRIVE guidelines recommended for reporting animal research

Laboratory animals	Adult homing pigeons (Columba livia) male and female, between one and four years of age.
Wild animals	This study did not involve wild animals.
Field-collected samples	This study did not involve field-collected samples.
Ethics oversight	All experiments were performed according to the principles regarding the care and use of animals adopted by the German Animal Welfare Law for the prevention of cruelty to animals as suggested by the European Communities Council Directive of November 24, 1986 (86/609/EEC) and were approved by the animal ethics committee of the Landesamt für Natur, Umwelt und Verbraucherschutz NRW, Germany.

Note that full information on the approval of the study protocol must also be provided in the manuscript.
